# Ex Vivo Characterization Studies Identify Candidate Therapies for the Individualized Care of *NF2*-Related Schwannomatosis

**DOI:** 10.3390/cancers18081209

**Published:** 2026-04-10

**Authors:** Ethan W. Hass, Anna Nagel, Alexandra J. Scott, Robert Allaway, Haley M. Hardin, Hollie M. Hayes, Lenna Huelbes, Alexander W. Sutton, Sofia A. Oliveira, Michelle Pei, Fred F. Telischi, John Ragheb, McKay McKinnon, Ziad Khatib, Mislen Bauer, Christine T. Dinh, Cristina Fernandez-Valle

**Affiliations:** 1 Burnett School of Biomedical Sciences, College of Medicine, University of Central Florida, Orlando, FL 32827, USA; 2 Sage Bionetworks, Seattle, WA 98121, USA; 3 Department of Otolaryngology, Miller School of Medicine, University of Miami, Miami, FL 33136, USA; 4 Division of Neurosurgery, Nicklaus Children’s Hospital, Miami, FL 33155, USA; 5 Division of Pediatric Plastic and Reconstructive Surgery, Nicklaus Children’s Hospital, Miami, FL 33155, USA; 6 Division of Hematology and Oncology, Nicklaus Children’s Hospital, Miami, FL 33155, USA; 7 Division of Clinical Genetics and Metabolism, Nicklaus Children’s Hospital, Miami, FL 33155, USA

**Keywords:** NF2, schwannomatosis, schwannoma, fimepinostat, dasatinib, brigatinib, neratinib, BMS-986158, drug sensitivity screens, spheroids

## Abstract

**Background/Objectives**: *NF2*-related schwannomatosis (*NF2*-SWN) is a genetic tumor predisposition syndrome of the nervous system caused by pathogenic variants in *NF2* encoding the merlin tumor suppressor. Truncating variants in *NF2* cause severe phenotypes with higher tumor burden, early mortality, and a lifetime need for multiple surgeries due to lack of medications that control schwannoma growth. **Methods**: We developed a functional precision medicine (FPM)-inspired workflow to identify drug sensitivities in cells isolated from a pediatric severe *NF2*-SWN patient’s spinal and peripheral schwannomas. Transcriptomic profiling, high-content drug sensitivity assays, tissue and isolated cell immunostaining, flow cytometry, and capillary-based immunoblotting were used to study the available tissues. **Results**: Aberrant merlin-dependent pathway expression was conserved between the spinal schwannoma and its cultured primary cells. Drug sensitivity screens in 2- and 3-dimensional formats revealed cytotoxic effects of fimepinostat in primary cells; dasatinib with brigatinib was the most effective cytostatic combination. Ineffective therapies attempted in the patient were also ineffective ex vivo. **Conclusions**: These data support the idea of using the FPM workflow to improve and individualize the standard of care for severe *NF2*-SWN patients using surgical samples.

## Simple Summary

*NF2*-related schwannomatosis (*NF2*-SWN) is a genetic syndrome that causes nervous system tumors. All *NF2*-SWN patients develop schwannomas in the nerves responsible for hearing and balance from both ears. Patients with severe *NF2*-SWN develop additional schwannomas and brain and spinal cord tumors, with no effective therapies. Patients attempt off-label drugs but eventually need surgical intervention. Resected tissues could be used as models for a patient’s growing tumors. Thus, we streamlined methods to study cells grown from a pediatric *NF2*-SWN patient’s spinal and peripheral schwannomas. This use of valuable surgical specimens could inform individualized care for *NF2*-SWN.

## 1. Introduction

*NF2*-related schwannomatosis (*NF2*-SWN), a genetic predisposition for developing benign nerve tumors of Schwann cell origin, is caused by germline pathogenic variants in *NF2,* which encodes the merlin tumor suppressor [1,2]. The hallmark of *NF2*-SWN is bilateral vestibular schwannomas (VS) on cranial nerve VIII with subsequent hearing loss [3]. However, more severe presentations have spinal and peripheral schwannomas as well as meningiomas and ependymomas, which cause additional loss of neurological function and reduce life expectancy [4]. The genotype–phenotype correlation and predictive factors for *NF2*-SWN severity have been described [5,6,7]. Truncating *NF2* variants cause particularly severe phenotypes and are often diagnosed in early childhood [8,9]. Time of diagnosis is the most predictive factor for early mortality in *NF2*-SWN patients [10]. Unilateral VS can also develop spontaneously in patients without *NF2*-SWN. Patients with hearing loss due to unilateral VS undergo surgical resection or radiotherapy, both of which carry additional risks of permanent hearing loss and facial nerve damage [11]. Patients with *NF2*-related VS have a comparatively higher risk of facial nerve damage during surgery [12], and possible secondary malignancies post-radiotherapy [13]. Surgical resection of *NF2*-related non-VS and meningiomas also carries risk of severe neurological dysfunction and death [14,15]. Pediatric and young adult patients with severe *NF2*-SWN would benefit from medical therapies that eliminate the need for repeated surgical interventions. 

To date, of nine drugs evaluated in ten clinical trials for *NF2*-SWN, none have promoted lasting target tumor volume regression (≥20%) by magnetic resonance imaging (MRI) in more than 30% of evaluable participants [16,17,18,19,20]. Two ongoing *NF2*-SWN clinical trials target focal adhesion kinase (FAK; NCT04374305, NCT04283669) which is one of many aberrantly activated signaling molecules that promote Schwann cell proliferation and survival upon merlin loss of function [21,22]. Current strategies for schwannoma growth control provide temporary benefits at best. Bevacizumab, an anti-vascular endothelial growth factor (VEGF) monoclonal antibody is used routinely to stabilize VS in responding patients. However, rebound tumor growth occurs when bevacizumab is terminated due to adverse events [23]. Patients who undergo VS resection after prolonged bevacizumab treatment also have an increased risk of intraoperative hemorrhage [24]. It is evident that novel pharmacologic approaches to schwannoma management are needed.

Because primary human schwannoma cells proliferate poorly in culture, preclinical studies have relied on mouse schwannoma cells, allografts, and genetically engineered mouse (GEM) schwannoma models to identify drug candidates. Drug efficacy is then confirmed in HPV E6/E7-immortalized VS cells (HEI-193) [25] or *hTERT/mCDK4* immortalized Schwann cells with *NF2* inactivation [26,27,28], both of which inaccurately model benign schwannoma cells in a patient. To address this limitation, we generated non-immortalized schwannoma model cells amenable to high-throughput screening campaigns (HS01, HS02) [29,30,31,32]. We then developed methods for isolating schwannoma cells from live-preserved tumor pieces and used high-content imaging techniques to evaluate candidate drugs and collect robust sensitivity data using fewer cells [33]. Herein, these methods were used to develop an ex vivo functional precision medicine (FPM) workflow for the individualized study of *NF2*-related schwannomas.

The successful use of FPM to study childhood cancers has been recently described [34]. Across multiple relapsed and refractory pediatric cancers, five of six patients treated with FPM-based recommendations experienced increased progression free survival (PFS) for up to 80 weeks compared with eight patients receiving standard of care who had PFS up to 20 weeks [34]. Other FPM studies have shown increased PFS in adult patients treated with interventions indicated by ex vivo sensitivity screens compared with standard of care [35,36]. We report the successful isolation and propagation of sufficient schwannoma cells from two resected non-VSs from a severe *NF2*-SWN patient to conduct curated drug sensitivity assays and validate drug target modulation. Cells from both tumors were sensitive to a combination of two FDA-approved multi-kinase inhibitors, dasatinib and brigatinib, which inhibit cell growth, and the dual HDAC/PI3K inhibitor, fimepinostat, a phase II investigational drug which promotes apoptotic cell death.

## 2. Materials and Methods

### 2.1. Clinical History and Radiographic Assessment of Tumors

Clinical data for this patient were obtained from the University of Miami and Nicklaus Children’s Hospital (NCH, Miami, FL). Electronic medical records were reviewed, including clinical history, physical examination findings, audiometric evaluations, imaging studies, and endoscopic laryngeal assessments. The intrathoracic portion of the T6–T7 schwannoma was measured using the institutional radiology software (Philips IntelliSpace Radiology Release 4.7 and Visage 7 Imaging version 7.1.19.675) by identifying the maximal linear dimension in the anteroposterior direction on sagittal T1-weighted post-contrast or T2-weighted MRI of the thoracic spine. Right and left vestibular schwannomas were measured on T1-weighted post-contrast MRI of the brain and internal auditory canal, with the maximum transverse linear dimension obtained from the fundus of the internal auditory canal toward the cerebellopontine angle. Tumor measurements were tracked longitudinally. Hearing outcomes were assessed by calculating the pure tone average (PTA, dB HL) across 500, 1000, 2000, and 4000 Hz for all available audiograms, and word recognition scores were documented over time. A chronological timeline was constructed to summarize clinical events and disease courses.

### 2.2. Schwannoma Dissection and Schwannoma Cell Culture

Tissues were received, processed, and preserved in accordance with the University of Central Florida’s Institutional Review Board under protocols 00001428 and 00001973. Multiple areas of resected schwannoma were received the day following surgery and fragments were preserved in cryotubes (Thermo Fisher Scientific, Waltham, MA, USA) containing heat-inactivated fetal bovine serum (FBS, R&D Systems, Bio-Techne, Minneapolis, MN, USA) with 10% dimethyl sulfoxide (DMSO). For cell isolation, fragments were digested in Dulbecco’s Modified Eagle Medium (DMEM, Gibco, Thermo Fisher Scientific, Waltham, MA, USA) with 0.5 mg/mL collagenase (Thermo Fisher Scientific) and 2.5 mg/mL dispase II (Sigma-Aldrich, St. Louis, MO, USA), incubated at 37 °C and 5% CO_2_ for 2 to 8 h with periodic mechanical digestion. The isolated cells were washed in DMEM with 10% FBS and resuspended in complete Schwann cell medium (SCM, ScienCell, Carlsbad, CA, USA), plated on Corning CellBind plates coated with growth factor-reduced Matrigel basement membrane (60 µg/cm^2^, Corning, Corning, NY, USA), which contains predominantly laminin and collagen type IV. Multiple derivations of primary cells were isolated from NCH1 tissues to provide fresh cells for FPM experiments. VSA91 primary schwannoma cells were isolated from a pediatric VS. NCH2 primary cells were isolated from a type I neurofibromatosis patient’s resected cervical tumor. Immortalized BH1hTERT cells were isolated from a peripheral schwannoma associated with SWN not elsewhere classified and were immortalized using lentiviral *hTERT* (Cellomics Technology, Halethorpe, MD, USA). All isolated primary tumor cells were cultured in SCM on Matrigel-coated CellBind surfaces. ST88-14 malignant peripheral nerve sheath tumor cells were cultured in Eagle’s Modified Eagle Medium (EMEM, Gibco) with 10% FBS on uncoated CellBind surfaces. All drug treatments were prepared in each primary cell’s respective standard medium.

Human schwannoma model cells were grown on uncoated CellBind surfaces. The HS02 model cell line was generated from human fetal Schwann cells (ScienCell, HSC7228) with lentiviral *NF2*-shRNA (#75, Sigma-Mission, Sigma-Aldrich) knockdown as described [31]. The HS05 model cell line was generated from the *Cdk4/hTERT* immortalized ipn02.3 2λ adult human Schwann cell line (University of Florida, Gainesville, FL, USA) with *NF2* knockout by CRISPR/Cas9 with NF2sg1 (sequence: AAACATCTCGTACAGTGACA, provided by the Broad Institute, Cambridge, MA, USA). 

### 2.3. Tissue Preparation and Immunohistochemistry

Schwannoma tissues were rinsed with PBS and fixed in 4% paraformaldehyde (PFA, Thermo Fisher Scientific) for 24–48 h at 4 °C. Tissues were then rinsed with 70% ethanol, processed using the Leica ASP300 S automated vacuum tissue processor (Leica, Wetzlar, Germany) and were embedded in paraffin wax. Five µm transverse sections were collected onto Fisher Superfrost plus slides (Thermo Fisher Scientific) and stained by hematoxylin and eosin (H&E) or used for immunohistochemistry (IHC). For IHC, slides were deparaffinized with xylene, rinsed with 100% ethanol and serially rehydrated with 95% and 70% ethanol and distilled water, respectively. Heat-induced epitope retrieval was performed in a water bath at 98 °C in either Antigen unmasking solution (pH 6.0, Vector Labs, Newark, CA, USA) for 20 min and cooled in buffer for 30 min at room temperature or 10 mM Tris base, 1 mM EDTA, 0.05% Tween 20 pH 9.0 for 15–45 min and cooled in buffer for 30 min at room temperature. Endogenous peroxidase activity was blocked with BLOXALL solution (Vector Labs) for 10 min at room temperature then blocked for 1 h at room temperature using 10% normal goat serum (NGS, Vector Labs) in Tris-buffered saline + 5% Tween 20 (TBS-T). Primary antibodies were diluted in 10% NGS/TBS-T, applied to samples, and incubated in a humidity chamber overnight at 4 °C. Sections were washed with TBS-T, then an ImmPRESS HRP Anti-Rabbit or Anti-Mouse IgG Polymer Detection Kit (Vector Labs) was applied and incubated at room temperature for 30 min. Colorimetric stains were developed with ImmPACT DAB HRP substrate (Vector Labs) followed by a hematoxylin counterstain, cleared with ethanol and xylene, and mounted with VectaMount Express mounting medium (Vector Labs). Slide imaging was performed with a Keyence BZ-X800 microscope (KEYENCE, Itasca, IL, USA). All primary antibodies with dilutions are listed in Table S1.

### 2.4. High-Content Drug Screening and Incucyte Live Cell Confluence Analysis

One thousand cells were seeded in 20 μL of medium per well on a 384-well CellBind plate. Primary tumor cells were grown on plates coated with 60 µg/cm^2^ Matrigel. After incubation for 16–20 h, cells in duplicate wells were treated with a curated library of 18 drugs or drug combinations at 3× treatment doses in 10 µL of medium (final well volume = 30 µL). Confluence in each well was assessed every 4 h over a total of 96 h using the Incucyte system (version 2024B, Sartorius, Gottingen, Germany).

### 2.5. Tumor Spheroid Development and Drug Screens

Primary cells were seeded onto 384-well ultra-low attachment (ULA) round-bottom plates (SBio, Constantine, MI, USA) at 1500 cells per well in 50 µL phenol red-free (prf) SCM containing 2.5% prf Matrigel and allowed to form spheroids for three days. After three days, 25 µL prf SCM supplemented with 2.5% prf Matrigel-containing drugs at 4× treatment doses and 25 µL containing cleaved caspase-3/7 (CC-3/7) green fluorescent reagent (Sartorius) were added (final well volume = 100 µL). Spheroid growth and CC-3/7 signal was monitored continuously over 10 days in 6 h increments using the Incucyte microscope (Sartorius). Spheroid area and integrated green intensity were assessed every 6 h for a total of 10 days using the Incucyte system (version 2024B, Sartorius).

### 2.6. Violet Ratiometric Cell Death Assay

Primary NCH1A cells were seeded at 75,000 cells/well in Matrigel-coated 6-well CellBind plates, grown for 24 h, and treated with indicated drugs for 72 or 96 h. Cells were then harvested with 0.05% trypsin (Gibco), washed in PBS, and resuspended in Hank’s Balanced Salt Solution (HBSS) without Ca^+2^/Mg^2+^. The Violet Ratiometric Asymmetry Assay (Invitrogen, Carlsbad, CA, USA) was used per the manufacturer’s instructions. Apoptotic, live and dead cell populations were measured using the Cytoflex LX flow cytometer and analyzed by CytExpert software (version 2.4.0.28, Beckman Coulter, Brea, CA, USA). 

### 2.7. Cell Cycle Analysis

NCH1A cells were seeded at 150,000 cells per well in Matrigel-coated 6-well CellBind plates, incubated for 24 h, and treated with indicated drugs for 24 h. EdU (10 μM; Click-iT EdU kit; Molecular Probes, Thermo Fisher Scientific) was added during the last 3 h of treatment. Cells were harvested with 0.05% trypsin, stained with violet live/dead stain (Thermo Fisher Scientific), and permeabilized. Total DNA labeling with FxCycle Far Red stain (Thermo Fisher Scientific) was conducted according to manufacturer’s protocol. Cell populations in G0/G1, S, or G2/M phase were analyzed using the CytoFlex LX flow cytometer and analyzed by CytExpert software (version 2.4.0.28, Beckman Coulter). 

### 2.8. Immunofluorescence (IF)

Cells were grown in a 96-well format, fixed in 4% PFA for 10 min, and stored in HBSS with Mg^2+^/Ca^2+^ (Gibco) at 4 °C until ready for staining. Before staining, cells were permeabilized using 0.1% Triton X-100 (Sigma-Aldrich), washed with HBSS, and blocked in 10% NGS/HBSS for 30 min. Primary antibodies were diluted in 10% NGS/HBSS and added to the wells for overnight incubation at 4 °C. Alexa Fluor-conjugated secondary antibodies (Invitrogen) were diluted 1:200 in 10% NGS/HBSS and incubated with cells for 40 min at room temperature, followed by DAPI and Alexa-Fluor 633-conjugated phalloidin (Invitrogen) counterstains for 10 min. Cells were post-fixed in 4% PFA for 5 min and stored in 100 μL HBSS per well. All wells were imaged using the Image Xpress PICO by Molecular Devices (San Jose, CA, USA) and multichannel images processed using ImageJ (1.54g). All primary antibodies with dilutions are listed in Table S1.

### 2.9. Jess Automated Immunoblotting

Total protein extracts from primary NCH1A cells were extracted using radioimmunoprecipitation (RIPA) buffer (Cell Signaling Technology, Danvers, MA, USA) supplemented with Halt protease and phosphatase inhibitor cocktail (Thermo Fisher Scientific). Protein was isolated and quantified using the detergent compatible (DC) protein assay (BioRad, Hercules, CA, USA). Chemiluminescence-based capillary electrophoresis assays were performed per manufacturer’s protocol (Jess Simple Western; ProteinSimple, Bio-Techne), 2.0–8.8 pg of protein was loaded per capillary in 40 nL. All primary antibodies with dilutions are listed in Table S1. Anti-rabbit and anti-mouse secondary horseradish peroxidase (HRP) antibodies (Bio-Techne) were used to label proteins. The ProteinSimple RePlex module for Jess was used to strip and reprobe each capillary for additional markers. Protein expression was quantified using Compass for SW (version 6.0.06, ProteinSimple) and normalized first to vinculin or β-actin loading controls, then to DMSO-treated controls for the respective treatment times. 

### 2.10. Sample Preparation for Transcriptomics

Tumor tissues were lysed using TRIzol (Thermo Fisher Scientific), and RNA was purified using the Zymo Direct-zol RNA isolation kit (Zymo Research, Irvine, CA, USA). Samples were sent in two batches to Novogene (Beijing, China) for mRNA sequencing. The first batch included four replicates of NCH1A solid schwannoma tissues and four replicates of HS02 and HS05 cells. The second batch included two samples each from P1 and P2 NCH1A.5 primary cells with four replicate HS02 samples for batch correction. All cells were in cultured 6-well CellBind plates for 48–72 h before extraction. Samples were sequenced using the Illumina NovaSeq X Plus (Illumina, San Diego, CA, USA) with 20 million paired reads per run. Raw fastq files were processed using the nf-core/rnaseq (v3.12.0) pipeline and quantified using salmon (v1.10.1) [37]. For genes with at least 10 total reads across samples, DESeq 2 (v1.46.0) [38] was applied to call differentially expressed genes (Benjamini-Hochberg adjusted *p*-value < 0.05). For comparisons with samples from multiple sequencing batches, we included the batch as a variable in the DESeq design formula. DEseq was used to perform principal component analysis (PCA) for the visualization of samples. GSVA R-package (v2.0.7) [39] was used to perform gene set variation analysis (GSVA) on all samples with the Kyoto Encyclopedia of Genes and Genomes (KEGG) pathway gene set [40,41,42]. Gene set enrichment analysis (GSEA) was performed, and enrichment plots were generated with the fgsea R-package (v1.32.4) [43] for the comparison between NCH1A schwannoma samples and cultured HS02 cells. 

### 2.11. Statistical Analysis

Graphs and statistical analyses were generated using GraphPad Prism version 10.5.0. High-content screens tested two or greater replicate wells per condition. Cell populations in flow cytometric analyses were compared using two-way ANOVA with Fisher’s least significant difference test. Normalized protein expression by capillary-based immunoblot were compared using repeated measures one-way ANOVA with Geisser–Greenhouse correction. Mean nuclear intensity and percent positive area by IF were compared using the Kruskal–Wallis test with the uncorrected Dunn’s test in 12 replicate regions of interest.

## 3. Results

### 3.1. Clinical Timeline

**Figure 1:**
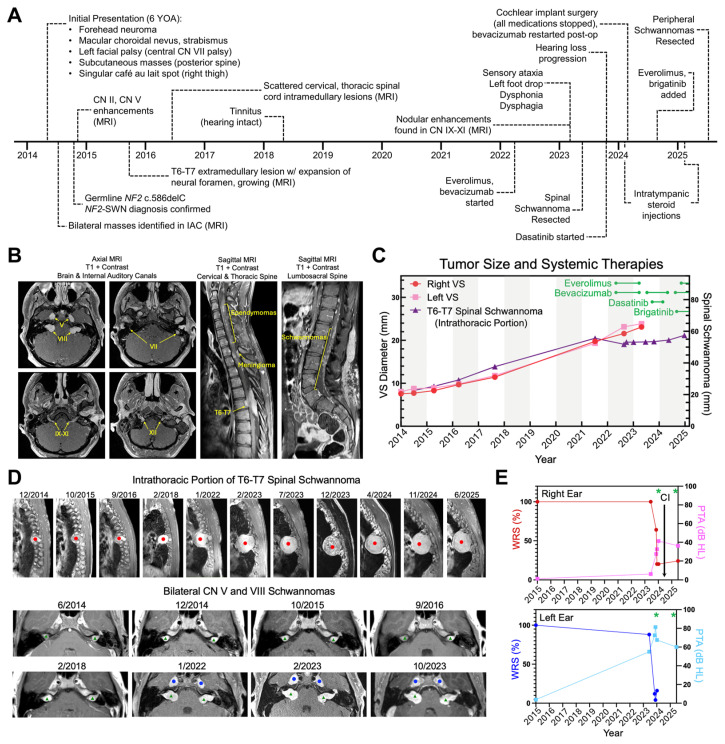
Tumor growth and hearing loss progression despite systemic therapy. (**A**) Clinical history for FPM pilot *NF2*-SWN patient (NCH1) who provided NCH1A and NCH1B schwannoma tissues for study. YOA: years of age. (**B**) MRI of schwannomas in cranial nerves V, VII, VIII, IX, X, XI, and XII, ependymomas in cervical spine (“string of pearls”), meningiomas in thoracic spine, T6–T7 spinal schwannoma (extending through vertebral foramen and intrathoracic space), and multiple schwannomas in the lumbosacral spine. (**C**) Tumor volume measurements for bilateral VS and intrathoracic portion of T6–T7 spinal schwannoma. (**D**) Progressive tumor growth on sagittal MRI for intrathoracic T6–T7 spinal schwannoma (red circle) and axial MRI for the bilateral CN V (blue circles) and bilateral CN VIII schwannomas (green triangles). (**E**) Word discrimination score (WRS) and pure tone average (PTA) in right and left ears. Oral steroids and intratympanic steroid injections (green asterisk) did not improve hearing in the right ear, and right cochlear implantation (CI) was performed when indicated. The PTA of the left ear remains stable on everolimus, bevacizumab, brigatinib and 2 rounds of steroids.

Figure 1A summarizes the clinical progression of the patient prior to resections of a spinal schwannoma in 2023 and multiple peripheral schwannomas in 2025. The patient (NCH1) was diagnosed with *NF2*-SWN at six years of age following removal of a subcutaneous neuroma on the forehead. At initial presentation, he had a left facial nerve palsy, strabismus, several palpable masses over the lumbar and thoracic spine, and a single café au lait spot on the right thigh. Brain MRI identified bilateral enhancing masses in the internal auditory canal consistent with bilateral VS on cranial nerve VIII. The genetic diagnosis of de novo *NF2*-SWN was made when lymphocyte sequencing identified an *NF2* frameshift pathogenic variant (c.586delC). Serial brain and spine MRIs tracked the growth of several tumors over the next decade, including lesions involving cranial nerves III, V, VII, and IX–XI as well as several tumors along the cervical, thoracic, and lumbosacral spine (Figure 1B–D). In 2022, the patient developed sensory ataxia, unsteady gait, left foot drop, and sensory deficits along the posterior thigh and lateral calf from a growing intradural extramedullary spinal mass with expansion of the T6–T7 vertebral foramina. He was started on bevacizumab (650 mg I.V.) every three weeks (Q3 weeks) and everolimus (5 mg P.O.) daily (QD); his condition did not improve with six months of treatment. In early 2023, the medications were discontinued in preparation for surgery. A T4–T7 laminectomy was performed; the tumor was debulked resulting in mild functional recovery and a stable intrathoracic mass on MRI (Figure 1D). Spinal tumor fragments were received and named NCH1A and used for ex vivo FPM studies. The patient was restarted on bevacizumab in August 2023 and dasatinib was briefly added (100 mg P.O., QD for 3 months) in late 2023 when he began to experience hearing loss in the left ear (Figure 1E). In early 2024, he developed progressive bilateral hearing loss that failed medical management with oral and intratympanic steroid injections (Figure 1E); all medications were temporarily stopped for right cochlear implant surgery. The patient also experienced worsening dysphonia, was found to have a left vocal cord paralysis, and received a left vocal fold injection with calcium hydroxylapatite. He was restarted on bevacizumab after his procedures. He reported worsening function in cranial nerves VII–XII. Brigatinib (180 mg P.O., QD) and intermittent everolimus (7.5 mg P.O., QD) were added in the latter half of 2024 and stopped in mid-2025 for resection of five painful subcutaneous masses on his trunk. These masses were received and collectively named NCH1B; one sample was used for additional ex vivo studies.

### 3.2. NCH1 Tumor Genetics and Histology Are Consistent with Schwannoma

**Figure 2:**
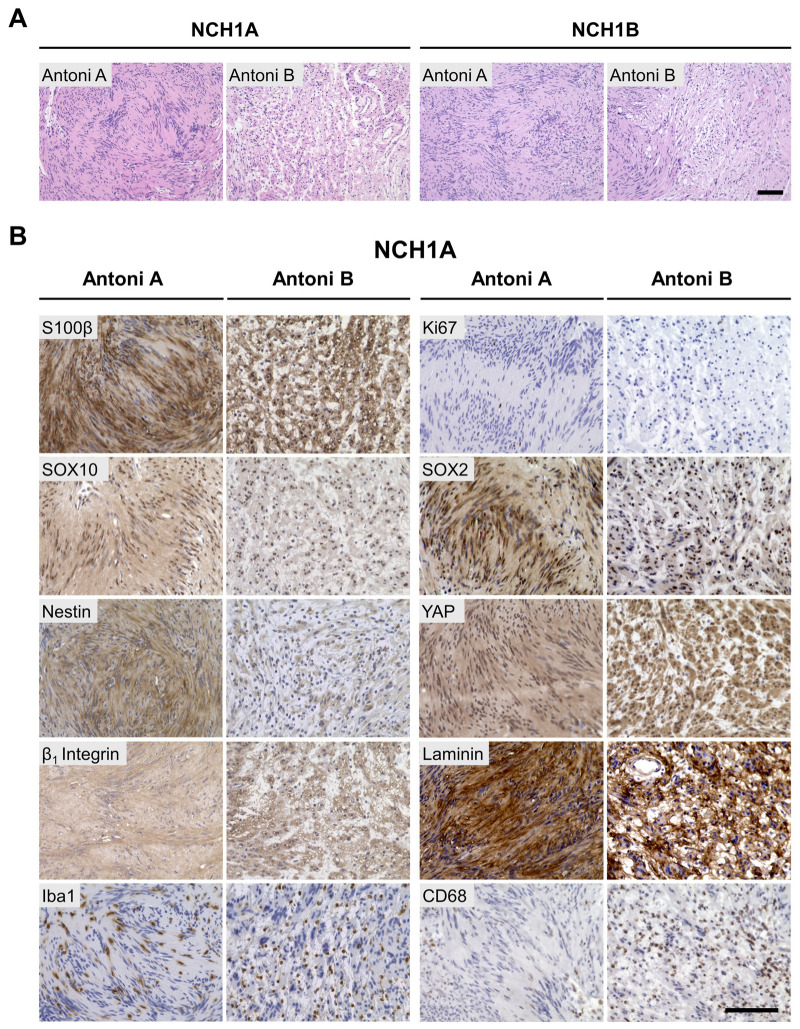
NCH1A and NCH1B histology demonstrates classic schwannoma architecture, low proliferative index, and expression of Schwann and schwannoma cell markers. (**A**) NCH1A and NCH1B H&E stains with characteristic Antoni A and B histomorphometric regions and Verocay bodies with nuclear palisading in Antoni A regions. (**B**) Representative IHC staining of indicated markers in NCH1A. Scale bar = 100 μm.

Spinal NCH1A and peripheral NCH1B schwannoma fragments (Figure S1A,B) were sent for clinical pathology and to the authors for FPM studies. Clinical pathology confirmed the diagnosis of schwannoma in both tissues. NCH1B was further distinguished as a plexiform schwannoma with a multinodular growth pattern. Genomic analysis of NCH1A tumor tissue reported a p.R196fs pathogenic variant identical to the patient’s germline mutation and copy neutral chromosome 22q loss of heterozygosity. There were no other large chromosomal alterations, copy number variants, or microsatellite instabilities (mutational burden = 3.4 mutations/Mb). NCH1A tissue was tested using a panel of 500 cancer-related genes and no changes other than for *NF2* were observed. NCH1B tissue contained the same p.R196fs variant with chromosome 22q loss of heterozygosity, and an otherwise normal genome (mutational burden = 2 mutations/Mb). NCH1A and NCH1B exhibited characteristic schwannoma architecture including Antoni A regions with densely packed cells in Verocay formations and loose Antoni B regions (Figure 2A). Immunohistochemistry (Figure 2B and Figure S1C) revealed scant Ki67 positivity in both histomorphometric regions, and positivity for S100β, SOX10, SOX2, nestin, and YAP with mixed nuclear positivity in both Antoni A and B regions. Additional markers relevant to Schwann cell biology were evaluated, including β1 integrin and its substrate, laminin, both of which were positive. The macrophage markers Iba1 and CD68 were present in both tumors.

### 3.3. Primary NCH1 Schwannoma Cells Express Characteristic Schwannoma Cell Markers

Primary cells were digested from both NCH1A and NCH1B tissues for characterization and drug screening. Within one day of isolation, primary cells from both tumors developed a spindle-like morphology with long, thin processes. NCH1A cells retained this morphology and proliferative capacity for up to eight passages (Figure S2A). NCH1B cultures, however, had a notable shift in cellular morphology by passage three (P3; Figure S2B). 

**Figure 3:**
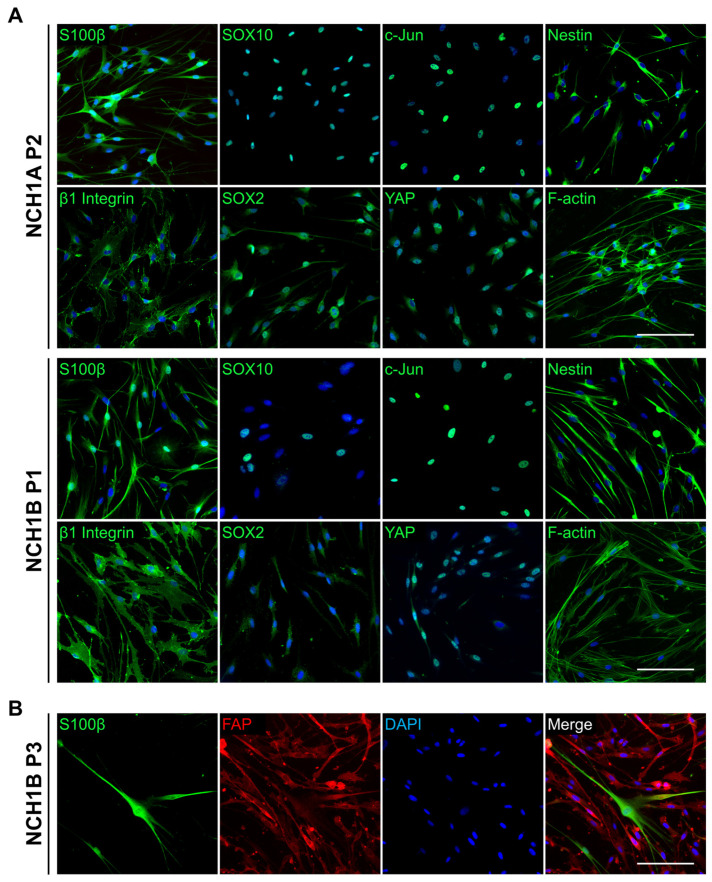
NCH1 spinal and peripheral schwannoma cells express characteristic schwannoma markers. (**A**) Representative IF staining for key Schwann and schwannoma cell markers in isolated NCH1A and NCH1B cells at indicated passages (P). (**B**) IF co-staining for Schwann cell marker S100β (green) and fibroblast marker FAP (red) in NCH1B cells at P3. Scale bars = 100 μm.

Primary cells isolated from NCH1A and NCH1B were characterized by IF using a panel of molecular markers. Cells from all derivations were positive for key Schwann/schwannoma cell markers: S100β, SOX10 (mixed nuclear positivity), and β1 integrin; Schwann cell repair-like state markers: c-Jun (mixed nuclear positivity) and cytoplasmic SOX2; and tumor cell markers: cytoplasmic nestin and YAP (Figure 3A and Figure S3). Both NCH1A and NCH1B cultures contained cells that co-expressed the macrophage marker CD68 with S100β (Figure S4A). However, no cells were positive for the macrophage marker Iba1 (Figure S4B). Cultures of NCH1B cells were predominantly S100β-positive at P1 but were predominantly S100β-negative and fibroblast activation protein (FAP)-positive at P3 (Figure 3B). Therefore, all subsequent experiments using NCH1B cells were conducted at P1. NCH1A cells retained S100β-positivity up to P4, the highest passage evaluated.

### 3.4. Merlin-Related Tumorigenic Pathways Are Conserved in NCH1A Solid Tissue, Primary Schwannoma Cells, and Human Merlin-Deficient Schwannoma Model Cells

**Figure 4:**
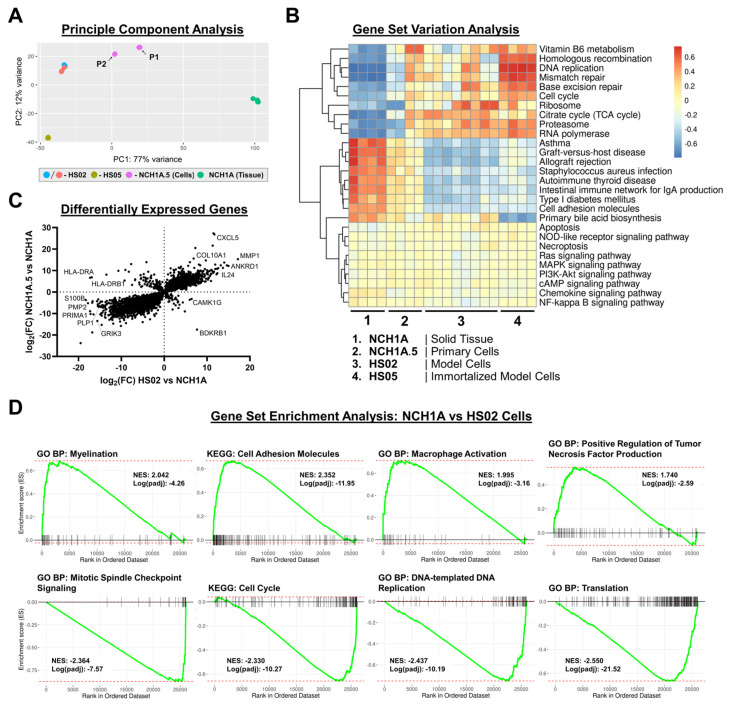
Primary schwannoma cells retain aberrant expression of merlin-dependent pathways but lack expression of immune-related pathways observed in tumor samples. (**A**) Principal component analysis comparing gene expression profiles of NCH1A solid schwannoma pieces, NCH1A.5 primary schwannoma cells (P1 and P2), and non-immortalized (HS02) and immortalized (HS05) human merlin-deficient schwannoma model cells. (**B**) Gene set variation analysis (GSVA) heatmap for indicated KEGG pathways in all samples. Pathways selected from the top 50 most- and least-variable pathways (Figure S5A). Color scale indicates GSVA enrichment score. (**C**) Fold change correlation plot for significant differentially expressed genes in patient-derived NCH1A.5 cells versus NCH1A tissue and human schwannoma model cells (HS02) versus NCH1A tissue. Both axes show log_2_ (fold change) in bulk mRNA expression. The bottom left quadrant represents genes with higher expression in solid tumor tissues compared with both model and primary cells in vitro. (**D**) Selected gene set enrichment (GSEA) plots comparing indicated pathway enrichment in NCH1A tissues versus HS02 schwannoma model cells. Normalized enrichment scores (NES) and adjusted *p*-values are reported on each plot.

Four solid pieces from different regions of NCH1A, two primary cell passages (P1 and P2), and two human merlin-deficient model cell lines (HS02, HS05) underwent transcriptomic analyses. The HS02 cell line is derived from non-immortalized fetal human Schwann cells and has lentiviral *NF2* shRNA knockdown of merlin [33]. The HS05 cell line is an immortalized adult human Schwann cell expressing *Cdk4/hTERT* with *NF2* knockout by CRISPR/Cas9. Principle component analysis (PCA; Figure 4A) illustrated the shift in patient-derived schwannoma cell transcriptional profiles from solid tumor tissues toward cultured model cells with subsequent passages. Cultured cells generally clustered together versus solid tissues in PC1 (contributing to 77% of variance between all samples). Gene set variation analysis (GSVA) showed conservation of key merlin-dependent pathway signaling (Ras, MAPK, PI3K), chemokine signaling, inflammatory signaling (NF-κB, NOD-like receptor), and cell death-related pathways across all samples (Figure 4B). Immune-related pathways (ex: asthma, graft-versus-host disease, allograft rejection) were enriched in NCH1A tissues, whereas cell cycle and DNA replication pathways were enriched in cultured primary and model cells, especially in the immortalized HS05 model cells. Expanded GSVA comparing all samples using the KEGG 2021 human gene set are shown in Figure S5A. Differentially expressed genes (DEGs) upregulated in NCH1A tissues versus primary cells and the HS02 model cell line included those expressed in myelinating Schwann cells (*PMP2*, *PLP1*; Figure 4C). DEGs downregulated in NCH1A tissues versus primary and HS02 cells were those related to extracellular matrix remodeling (*COL10A1*, *MMP1*) and chemoattractant proteins that promote M2 macrophage polarization (*IL24*, *CXCL5*). Both *TERT* and *CDK4* genes were significantly upregulated in the immortalized HS05 cells compared with HS02 cells, as expected (Figure S5B). According to gene set enrichment analysis (GSEA), NCH1A tissues expressed higher levels of myelination, cell adhesion, macrophage activation, and tumor necrosis factor production pathways than HS02 cells. HS02 cells expressed higher levels of mitotic spindle checkpoint, cell cycle, DNA replication, and translation pathways than tumor tissue (Figure 4D).

### 3.5. Fimepinostat and Dasatinib Were the Most Effective Monotherapies; BMS-986158 with Trametinib Was the Most Effective Combination Therapy in NCH1A Cells

**Figure 5:**
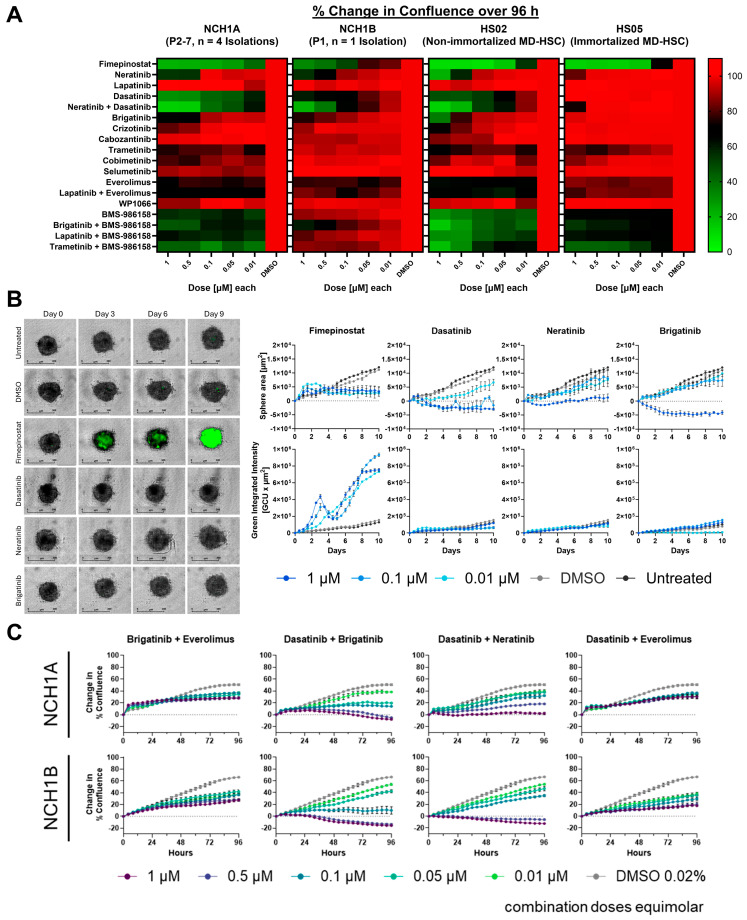
NCH1 Schwannoma cells are sensitive to HDAC inhibition and combinations with SFK inhibition. (**A**) Heatmaps illustrate differences in well confluence versus DMSO controls after 96 h of treatment in high-content drug screens. MD-HSC = merlin-deficient human schwannoma model cell. NCH1A values are averaged across four unique primary cell derivations. (**B**) Normalized spheroid area and cell death (green integrated intensity) measured in NCH1A-derived spheroids over 10 days of treatment with indicated monotherapies at 0.1 μM. Area was normalized to the initial time of treatment for each spheroid. (**C**) Representative confluence curves for NCH1A and NCH1B cells treated with brigatinib and everolimus (previous therapy for NCH1B) and with dasatinib in combination with brigatinib, neratinib, and everolimus. Change in percent confluence was normalized to the time of initial treatment. All doses were screened in duplicate wells. SFK = Src family kinases.

Four primary cell derivations from NCH1A and one derivation from NCH1B were initially screened using a 384-well high-content assay with a curated panel of 18 *NF2*-SWN candidate drugs or combinations encompassing drugs in off-label use, in clinical trials for *NF2*-SWN, and other pre-clinical drugs (Table S2, five doses ranging 1 to 0.01 μM; Figure 5A and Figures S6 and S7A). The non-immortalized HS02 and immortalized HS05 merlin-deficient human schwannoma model cell lines were screened using the same curated drug panel for comparison. Difference in well confluence after 96 h of drug treatment versus DMSO-treated controls was the primary metric for drug efficacy. Each monotherapy was also tested in NCH1A-derived tumor spheroids with indicators for CC-3/7 to evaluate caspase-dependent cell death (Figure 5B and Figure S7B). We observed the following for monotherapies in order of most to least effective. Fimepinostat, a dual HDAC/PI3K inhibitor, reduced well confluence after 96h with 0.1 µM treatment compared with DMSO-treated controls by 76%, 50%, 92%, and 86% for NCH1A, NCH1B, HS02, and HS05 cells, respectively (Figure 5A). At 0.1 µM, fimepinostat also reduced NCH1A spheroid area compared with DMSO-treated controls by 15% over 10 days of treatment and CC-3/7 was detectable after 1 day of treatment (Figure 5B). At 0.1 µM, the Src family kinase (SFK)/ABL inhibitor dasatinib reduced confluence compared with DMSO-treated controls in both primary cells and HS02 cells, but not in HS05 cells (NCH1A = 58%, NCH1B = 29%, HS02 = 47%, HS05 = 0% reduction). Dasatinib was the most effective drug at reducing NCH1A spheroid area by 28% compared with DMSO-treated controls, although no CC-3/7 was detected. Neratinib, a pan-HER inhibitor, had a similar effect to dasatinib in reducing non-immortalized cell confluence compared with DMSO-treated controls at 0.5 µM (NCH1A = 46%, NCH1B = 29%, HS02 = 42%, HS05 = 1% reduction) and reduced NCH1A spheroid area by 13% at 0.1 µM compared with DMSO-treated controls. At 1 µM, the ALK inhibitor brigatinib was less efficacious at reducing primary cell confluence compared with DMSO-treated controls than dasatinib and neratinib (NCH1A = 34%, NCH1B = 25%, HS02 = 46%, HS05= 3% reduction); at 0.1 µM it reduced NCH1A spheroid area by 17% of DMSO-treated control size. At 1 µM, the mTOR inhibitor everolimus was less efficacious than brigatinib at reducing primary cell confluence compared with DMSO controls (NCH1A = 31%, NCH1B = 23%, HS02 = 63%, HS05 = 14% reduction); at 0.1 µM it reduced NCH1A spheroid area by 15% of DMSO-treated control size. At 1 µM, the MEK inhibitor trametinib was less efficacious than everolimus at reducing primary cell confluence compared with DMSO-treated controls (NCH1A = 25%, NCH1B = 5%, HS02 = 30%, HS05 = 20% reduction); at 0.1 µM it reduced NCH1A spheroid area by 6% of control size. Trametinib was the only drug other than fimepinostat to cause detectable CC-3/7 in NCH1A spheroids, although it was present only in the core between 1 and 6 days of treatment (Figure S7B). The bromodomain and extraterminal protein (BET) inhibitor BMS-986158 had cytostatic effects at 0.1 µM in NCH1A cells and both model cell lines, but did not meaningfully reduce confluence in NCH1B cells compared with DMSO-treated controls (NCH1A = 46%, NCH1B = 8%, HS02 = 56%, HS05 = 35% reduction); at 0.1 µM it caused NCH1A cells within the spheroids to elongate, increasing total area by 15% of control size. Thus, the initial monotherapy screens indicated that fimepinostat was the most effective cytotoxic compound in primary NCH1 cells and model human schwannoma cells. Dasatinib was the most effective cytostatic compound in primary NCH1 and non-immortalized model human schwannoma cells.

The initial screens also included combinations with the BET inhibitor BMS-986158 based on the results of previously published work [44]. Trametinib with BMS-986158 was the most effective of these combinations; at 0.1 µM each, it reduced NCH1A confluence compared with DMSO-treated controls (NCH1A = 56%, NCH1B = 16%, HS02 = 43%, HS05 = 55% reduction). Additional assays to detect cell death in both adherent NCH1A cells and NCH1A spheroids confirmed the mild cytotoxic effects of trametinib combined with BMS-986158 at 1 µM each (Figure S8). 

### 3.6. Dasatinib with Brigatinib Was the Most Effective Combination in NCH1A and NCH1B Cells in a Secondary Screen

Follow-up screens testing additional drug combinations in NCH1A and NCH1B cells evaluated the patient’s current small molecule drug regimen (brigatinib with everolimus) versus the most potent cytostatic monotherapy, dasatinib, with kinase inhibitors in clinical use (brigatinib, neratinib, and everolimus, Figure 5C). Of the combinations tested, dasatinib with brigatinib was the most effective; it reduced NCH1A and NCH1B well confluence compared with DMSO-treated controls by 91% and 93%, respectively, after 96 h treatment with 0.5 µM each. By comparison, brigatinib with everolimus at 0.5 µM each reduced NCH1A and NCH1B well confluence by 44% and 37%, respectively, over DMSO-treated controls. Multiple patient-derived cell cultures from other schwannomas and other nerve tumors (neurofibroma, malignant peripheral nerve sheath tumor) were also screened using modified versions of the FPM paradigm (summarized in Figure S9). Notably, fimepinostat reduced confluence compared with DMSO-treated controls by 60% or more at 0.1 µM in all patient-derived tumor cells treated for 96 h.

### 3.7. Fimepinostat Is Cytotoxic and Dasatinib, Trametinib, and BMS-986158 Are Cytostatic in NCH1A Cells

**Figure 6:**
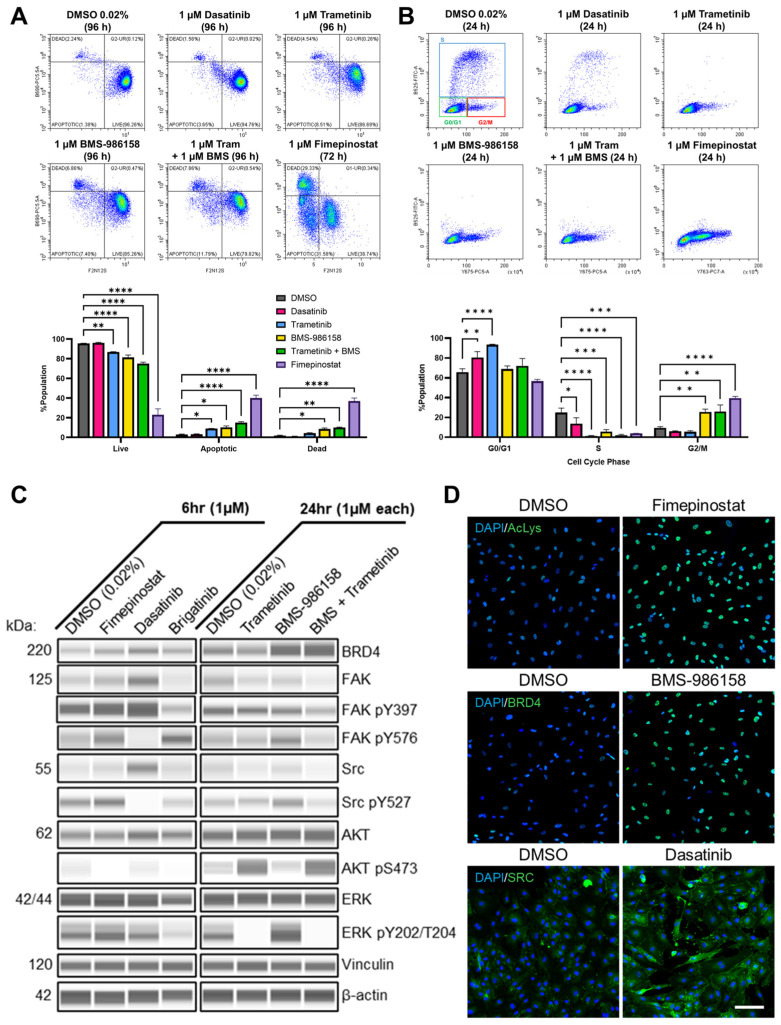
Targeting Src and MEK is associated with cell cycle arrest in NCH1A schwannoma cells and targeting HDAC, and MEK with BRD4 is associated with cell death. (**A**) Flow cytometric assessment of cell death (violet ratiometric assay) in NCH1A cells treated for 72 h (fimepinostat) and 96 h (trametinib, BMS-986158, trametinib + BMS-986158 equimolar; *n* = 3). (**B**) Flow cytometric assessment of cell cycle arrest (Click-iT EdU) in NCH1A cells treated for 24 h with indicated drugs. (**C**) Representative Jess capillary-based immunoblots for key drug targets in NCH1A schwannoma cell lysates after treatment with 1 µM each for indicated times. (**D**) Representative IF for indicated drug targets in NCH1A cells treated with top performing drug candidates for 24 h. Quantitation shows mean nuclear intensity and percent positive area (*n* > 6 images per condition). * *p* < 0.05, ** *p* < 0.01, *** *p* < 0.001, **** *p* < 0.0001. Scale bar = 100 µm. *n* = 3 biological replicates.

We selected fimepinostat, dasatinib, and the combination of trametinib with BMS-986158 for further evaluation in NCH1A cells. To measure cell death, we performed violet ratiometric flow cytometry assays in NCH1A cells treated with 1 µM for 72 h (fimepinostat) and 96 h (trametinib with BMS-9816158, alone an in equimolar combination; Figure 6A). Fimepinostat yielded a 37% dead cell population and a 40% apoptotic cell population by 72 h of treatment (*p* < 0.0001). The combination of trametinib and BMS-986158 yielded a 10% dead cell population (*p* = 0.0043) and a 15% apoptotic cell population (*p* < 0.0001) by 96 h of treatment. To measure cell cycle arrest, we performed flow cytometric analysis of EdU incorporation and total DNA content in NCH1A cells treated with 1 µM dasatinib, trametinib, BMS-986158, and the combination of trametinib with BMS-986158 for 24 h (Figure 6B). Dasatinib and trametinib increased the G0/G1 cell population compared with DMSO-treated controls from 66% to 80% (*p* = 0.0074) and to 93% (*p* < 0.0001), respectively. BMS-986158 induced a G2/M cell cycle arrest, with population increasing from 9% in DSMO-treated controls to 26% with treatment (*p* = 0.0035). When combined at 1 µM each, trametinib and BMS-986158 also increased the cell population in G2/M from 9% to 26% (*p* = 0.0029). These results demonstrate that, in primary NCH1A cells, fimepinostat is cytotoxic and dasatinib, trametinib, and BMS-986158 are cytostatic.

### 3.8. Fimepinostat, Dasatinib, Trametinib, and BMS-986158 Modulate Their Respective Targets in NCH1A Cells

Capillary-based immunoblots were performed to assess drug target modulation in NCH1A cells (Figure 6C and Figure S10A). At 1 µM for 6 h, fimepinostat decreased AKT phosphorylation at serine 473 (pS473) by 90% compared with DMSO-treated controls (*p* < 0.01). At 1 µM for 24 h, fimepinostat also increased nuclear lysine acetylation levels detected on IF by 175% (*p* < 0.0001) and nuclear BET protein (BRD4) by 19% (*p* < 0.0001; Figure 6D and Figure S10B) compared with levels in DMSO-treated controls. By 6 h of treatment at 1 µM, dasatinib decreased FAK pY576 by 53% (*p* < 0.05) compared with DMSO-treated controls; brigatinib increased FAK pY576 by 164% under the same conditions (*p* < 0.01). Neither dasatinib nor brigatinib significantly modulated FAK pY397 compared with DMSO. Across three biological replicates, brigatinib had a variable effect on FAK pY397 depending on passage number (ranging from P2 to P8). However, brigatinib reduced AKT pS473 and ERK pY202/T204 levels compared with DMSO-treated controls by 71% and 47%, respectively (*p* < 0.01). Dasatinib decreased Src pY527 at 6 h on immunoblot by 87% (*p* < 0.01) and increased total Src levels at 24 h on IF by 137% (*p* < 0.05) compared with DMSO-treated controls. An amount of 1 µM trametinib decreased ERK pY202/T204 at 24 h on immunoblot compared with DMSO with and without the addition of 1 µM BMS-986158 (89% reduction in combination, 93% reduction alone; *p* < 0.01), whereas 1 µM BMS-986158 monotherapy increased ERK pY202/T204 by 61% (*p* < 0.01). An amount of 1 µM trametinib alone increased AKT pS473 by 110% (*p* < 0.05) compared with DMSO. BMS-986158 treatment for 24 h increased levels of nuclear BRD4 protein on IF (20% versus DMSO, *p* < 0.0001), as did the combination of 1 µM BMS-986158 with 1 µM trametinib (15% versus DMSO, *p* < 0.0001). Collectively, these data validate molecular target modulation by fimepinostat (HDAC/PI3K), dasatinib (SFK/FAK), trametinib (MEK), and BMS-986158 (BRD4) in primary NCH1A schwannoma cells.

## 4. Discussion

Efforts to identify monotherapies for all tumor types and anatomical locations in *NF2*-SWN patients have been unsuccessful. Recently, an *NF2*-SWN clinical trial evaluated brigatinib and neratinib (NCT04374305). Brigatinib promoted volumetric responses in 4 of 38 target *NF2*-related tumors and 35 of 153 non-target tumors (median 10.4 months of treatment). Only one of the responding target tumors was a schwannoma; the other three were meningiomas [20]. Interim analysis for neratinib in twenty patients showed no radiographic response across 30 VS and 4 ependymomas, and responses in 6 of 17 non-VS and 3 of 18 meningiomas [45]. It is apparent that novel approaches are needed to address inter-patient tumoral heterogeneity while searching for effective *NF2*-SWN therapies.

Our drug screening method using resected patient tumor cells provides individualized data that could assist in clinical decision-making. The assay incorporates continuous live-imaging of isolated schwannoma cells on a laminin-rich substrate as is present in situ (Figure 2B). Phase images (Figure S6) provide insights into the timing, duration and morphological effects of the drugs tested. This is an improvement in depth over other FPM studies in solid and hematological malignancies that used single-timepoint viability assays (CellTiter Glo) to measure drug sensitivity in organoids, semi-adherent cultures, and suspended cells [34,35,36]. In our ex vivo high-content screens, we identified effective drugs that outperformed drugs in clinical use for *NF2*-SWN, including a combination prescribed for the patient that previously failed. 

Our data suggest that Src is superior to FAK as a druggable target for *NF2*-related schwannomas. Dasatinib was more effective in reducing cell confluence than the FAK inhibitors brigatinib and crizotinib in *NF2*-SWN clinical trials (NCT04374305, NCT04283669). Dasatinib is FDA-approved for Philadelphia chromosome-positive chronic myeloid leukemia. We also previously reported preclinical efficacy in patient-derived cells for dasatinib with the PI3K inhibitor omipalisib [33]. In NCH1A cells, dasatinib reduced FAK pY576 levels and promoted cell cycle arrest. Brigatinib did not reduce FAK pY576 levels nor promote cell cycle arrest. Neither drug significantly modulated FAK pY397 levels. FAK Y397 is a binding site for Src, one that allows Src to phosphorylate FAK Y576, thereby activating FAK [46]. Dasatinib decreased Src pY527 levels in NCH1A cells, consistent with loss of its kinase activity [47,48]. Unfortunately, it was not possible to evaluate dasatinib’s efficacy in the patient due to early discontinuation after 3 months for cochlear implant surgery.

The combination of dasatinib with brigatinib was the most efficacious in schwannoma cells isolated from both NCH1 tumors, evidenced by decreased growth of >90% compared with DMSO-treated controls. Dasatinib and brigatinib are both FDA-approved for other indications and have tolerable safety profiles [20,49]. The Synodos for NF2 consortium identified dasatinib with brigatinib as being among the most potent combinations in a large screening campaign using human schwannoma model cells (HS01) and their isogenic, merlin-intact parental cells (HS11). Dasatinib differentially inhibited SFKs and ABL1/2, as well as EphA2/B2, and PDGFRβ, and other kinases in a 24 h kinome assay [32]. Using the same method, brigatinib differentially inhibited Fer and FAK, as well as Cdc42-asociated kinase and aurora kinase among others [32]. The major target of brigatinib, ALK, is not expressed in schwannoma cells. Neither monotherapy induced significant changes in gene expression. However, the combination of 0.6 µM dasatinib and 1 µM brigatinib for 24 h induced 1397 DEGs in HS01 cells and 1032 DEGs in HS11 cells versus untreated controls, with 679 shared DEGs between the two cell types. Pathway analysis by Chang et al. revealed decreased NIK/NFκB- and tumor necrosis factor-mediated signaling in HS01 cells but not HS11, suggesting a possible inflammatory response in merlin-deficient schwannoma model cells [32]. Dose-limiting toxicity in genetically engineered *NF2*-SWN model mice was attributed to brigatinib in the dasatinib/brigatinib combination prevented accurate evaluation [32], and the combination was not advanced into clinical trial. Brigatinib combined with everolimus had a mild cytostatic effect in our 4-day high-content assay and was ineffective in controlling growth of the patient’s resected peripheral tumors over 6 months of treatment. The patient has been restarted on brigatinib with everolimus and continues bevacizumab infusions for control of cranial and spinal tumors post-surgery. 

Fimepinostat was the only monotherapy to promote cell death in all derivations of primary NCH1 cells. Moreover, it induced caspase cleavage in the spheroid assay and increased apoptotic cell count during flow cytometric analysis (Figure 5B and Figure 6A). As expected, the HDAC/PI3K inhibitor decreased AKT pS473 and increased nuclear levels of acetylated lysine and the BET protein BRD4 [50]. Previously, we demonstrated that CUDC-907, a generic form of fimepinostat, promoted cell death in five primary VS cell cultures and that drug sensitivity correlated with higher levels of phosphorylated HDAC2 [51]. In Schwann cells, HDACs regulate both development and response to injury through master transcriptional regulators like SOX10, SOX2, and NF-κB [52,53,54]. Thus, HDAC inhibition has numerous downstream consequences that can lead to schwannoma cell death and tumor regression. Fimepinostat was recently evaluated in pediatric and young adult patients with solid and CNS tumors (NCT03893487) and had a tolerable safety profile [55]. 

BET inhibitors have demonstrated preclinical efficacy in mouse and human schwannoma model cells and murine allograft models [28,44,56]. Furthermore, triple combinations of BET inhibitors with MEK inhibitors and CDK inhibitors have demonstrated efficacy in related malignant peripheral nerve sheath tumor mouse models [57]. We observed differences in drug sensitivity to BMS-986158 between the spinal and peripheral schwannoma cells. BMS-986158 was effective in NCH1A cells but ineffective in NCH1B cells. It is unclear whether the 10 months of treatment with combinations of brigatinib, everolimus, and bevacizumab prior to peripheral schwannoma resection or tumor location contributed to the differences in BMS-986158 sensitivity. We do not suspect the 3-month treatment with dasatinib contributed to the differences in sensitivity because the patient had not taken dasatinib for 18 months before the tumors were resected. BMS-986158 has also been evaluated in clinical trials as a combination therapy for myelofibrosis with a tolerable safety profile [58]. Trametinib was the most effective drug tested in combination with BMS-986158, as previously reported [44], and is FDA approved with a tolerable safety profile [59]. 

The transcriptomic analyses of human model versus patient-derived schwannoma cells and tumor tissue revealed low variance in expression of genes associated with aberrant merlin-dependent proliferation, and survival pathways. This validates the use of non-immortalized model and patient derived schwannoma cells to screen for drugs that inhibit deregulated signaling due to merlin loss. Recently, a single cell transcriptomic study revealed conservation of 6 transcriptional metaprograms across 22 schwannomas from distinct anatomical locations [60]. These metaprograms were associated with cellular stress, myelin, immune signaling (MHC class II and interferon), glycolysis/hypoxia, and extracellular matrix maintenance. The NCH1 solid tumor tissues similarly expressed myelination, cell adhesion, and macrophage activation-related pathways, which were diminished in cultured cells. A loss of immune-related pathway expression in primary cells compared with tumor tissue reflects a loss of monocytes and macrophages during culture and was corroborated by a lack of Iba1 staining (Figure S4B). CD68 expression was observed but has been reported previously in cultured Schwann cells [61] and does not necessarily indicate the presence of immune cells. We also observed conservation of Schwann and schwannoma cell marker expression between tissue IHC and cell IF despite the faster proliferation rate of cultured schwannoma cells than *in situ*. The limitations of increased primary schwannoma cell proliferation and immune cell deficiency will be addressed in future experiments. It should also be noted that merlin-intact Schwann cells were not included in the transcriptomic comparison. Because wild-type Schwann cell monocultures have little phenotypic similarity to mature, post-mitotic, myelinating and non-myelinating Schwann cells, such controls would not accurately reflect a healthy Schwann cells transcriptomic profile.

## 5. Conclusions

These data demonstrate that long-term preservation of resected schwannoma tissues and subsequent isolation of primary cells for FPM studies is feasible. Moreover, individualized ex vivo drug sensitivity screens using this workflow for a patient with severe *NF2*-SWN refractory to current medical treatments indicated candidate therapies that would not have otherwise been explored. Previous drug regiments that failed in this patient were also ineffective in ex vivo screens. Without such studies, this patient has limited alternative therapeutic options to prevent or eliminate the need for future surgeries. Non-*NF2*-SWN associated with germline pathogenic variants in *SMARCB1* and *LZTR1* that depend on surgical management of multiple schwannomas could also benefit from FPM studies. Finally, our transcriptomic analyses revealed that non-immortalized human schwannoma model cells had similar gene expression profiles and drug sensitivity profiles to primary schwannoma cells. Together, these human schwannoma cell models could provide more impactful preclinical data for all *NF2*-SWN subtypes.

## Data Availability

The original transcriptomic data analyzed and presented in the study are openly available in the NF Data Portal powered by Synapse at syn66279805.

## Supplementary Materials

The following supporting information can be downloaded at https://www.mdpi.com/article/10.3390/cancers18081209/s1, Table S1: Antibodies and dilutions used; Figure S1: NCH1A and NCH1B gross histology and NCH1B immunohistochemistry; Figure S2: Morphological appearance of primary NCH1 schwannoma cells; Figure S3: Individual channels for NCH1 schwannoma cell marker expression; Figure S4: Expression of immune markers by NCH1 schwannoma cells; Figure S5: Expanded analysis of NCH1A tissues and cultured schwannoma cells; Table S2: Drugs screened and target pathways; Figure S6: Morphological changes in NCH1 schwannoma cells with indicated drug treatments; Figure S7: Confluence changes and spheroid growth in NCH1 schwannoma cells with indicated drug treatments; Figure S8: Cell death measured by incucyte in NCH1A adherent cells and spheroids with fimepinostat and trametinib combined with BMS-986158; Figure S9: High-content drug screen results in other nerve tumor cells; Figure S10: Jess immunoblot and IF quantification in NCH1A schwannoma cells.

## Abbreviations

The following abbreviations are used in this manuscript:

BETs	Bromodomain and extraterminal proteins
BRD4	Bromodomain-containing protein 4
CC-3/7	Cleaved caspase 3/7
DEGs	Differentially expressed genes
DMEM	Dulbecco’s modified Eagle’s medium
FAK	Focal adhesion kinase
FBS	Fetal bovine serum
FPM	Functional precision medicine
GEM	Genetically engineered mouse
GSEA	Gene set enrichment analysis
GSVA	Gene set variation analysis
H&E	Hematoxylin and eosin
HBSS	Hank’s balanced salt solution
IF	Immunofluorescence
IHC	Immunohistochemistry
MD-HSC	Merlin-deficient human Schwann cell
MRI	Magnetic resonance imaging
NES	Normalized enrichment score
NF2-SWN	*NF2*-related schwannomatosis
PCA	Principal component analysis
PFA	Paraformaldehyde
prf	Phenol red-free
PTA	Pure tone average
SFK	Src family kinase
TBS	Tris-buffered saline
VEGF	Vascular endothelial growth factor
VS	Vestibular schwannoma
WRS	Word recognition score
