# Supplementary material for: Ex Vivo Characterization Studies Identify Candidate Therapies for the Individualized Care of NF2-Related Schwannomatosis
Source: Cancers (Basel). 2026 Apr 10;18(8):1209. doi: 10.3390/cancers18081209 (PMC13114645; doi:10.3390/cancers18081209)
Supplement: Supplementary file 1 [file cancers-18-01209-s001.zip › cancers-4189765-supplementary.pdf]

Table S1. Antibodies and Dilutions Used.

Marker	Vendor	Cat#	Dilution
**Immunohistochemistry**			
β1 Integrin	Epitomics	1798-1	1:500
CD68	Novus Bio	nbp2-32832	1:200
Iba1	Cell Signaling	17198	1:800
Ki-67	Abcam	ab16667	1:200
Laminin	Sigma	L9393	1:50
Nestin	Cell Signaling	33475	1:500
S100β	Novus Bio (NCH1A),<br>Abcam (NCH1B)	nbp2-45267 (NCH1A),<br>ab52642 (NCH1B)	1:1,000
SOX10	Abcam	ab155279	1:100 (NCH1A),<br>1:800 (NCH1B)
SOX2	Abcam	ab97959	1:100 (NCH1A),<br>1:800 (NCH1B)
YAP	Cell Signaling	14074	1:1,000
**Immunofluorescence**			
Acetylated lysine	Cell Signaling	9441	1:400
BRD4	Cell Signaling	63759	1:500
CD163	cell signaling	mab93498	1:300
CD68	Novus Bio	nbp2-32832	1:100
c-Jun	Cell signaling	9165	1:200
FAK	BD Biosciences	610087	1:50
Iba1	Cell Signaling	17198	1:100
Nestin	Cell Signaling	33475	1:100
S100β	Abcam (Rab IgG)	ab52642	1:100
S100β	Sigma (Mus IgG)	S2532	1:250
SOX2	abcam	ab97959	1:100
SRC	Cell Signaling	2109	1:50
YAP	Cell signaling	mab14074	1:200
β1 Integrin	Novus bio	MAB17781	1:100
**Jess Capillary-based Immunoblotting**			
AKT	Cell Signaling	2920S	1:50
AKT pS473	Cell Signaling	4058S	1:200
BRD4	Fortis Life Sciences	A301-985A100	1:20
ERK	Cell Signaling	4696	1:200
ERK pT202/Y204	Cell Signaling	4370S	1:50
FAK	Cell Signaling	13009	1:50
FAK pY397	Cell Signaling	8556P	1:100
FAK pY576	Cell Signaling	3281	1:100
SRC	Cell Signaling	2109S	1:100
SRC pY527	Cell Signaling	2105S	1:100
Vinculin	Cell Signaling	13901	1:4,000
β-actin	Cell Signaling	4970S	1:1,500

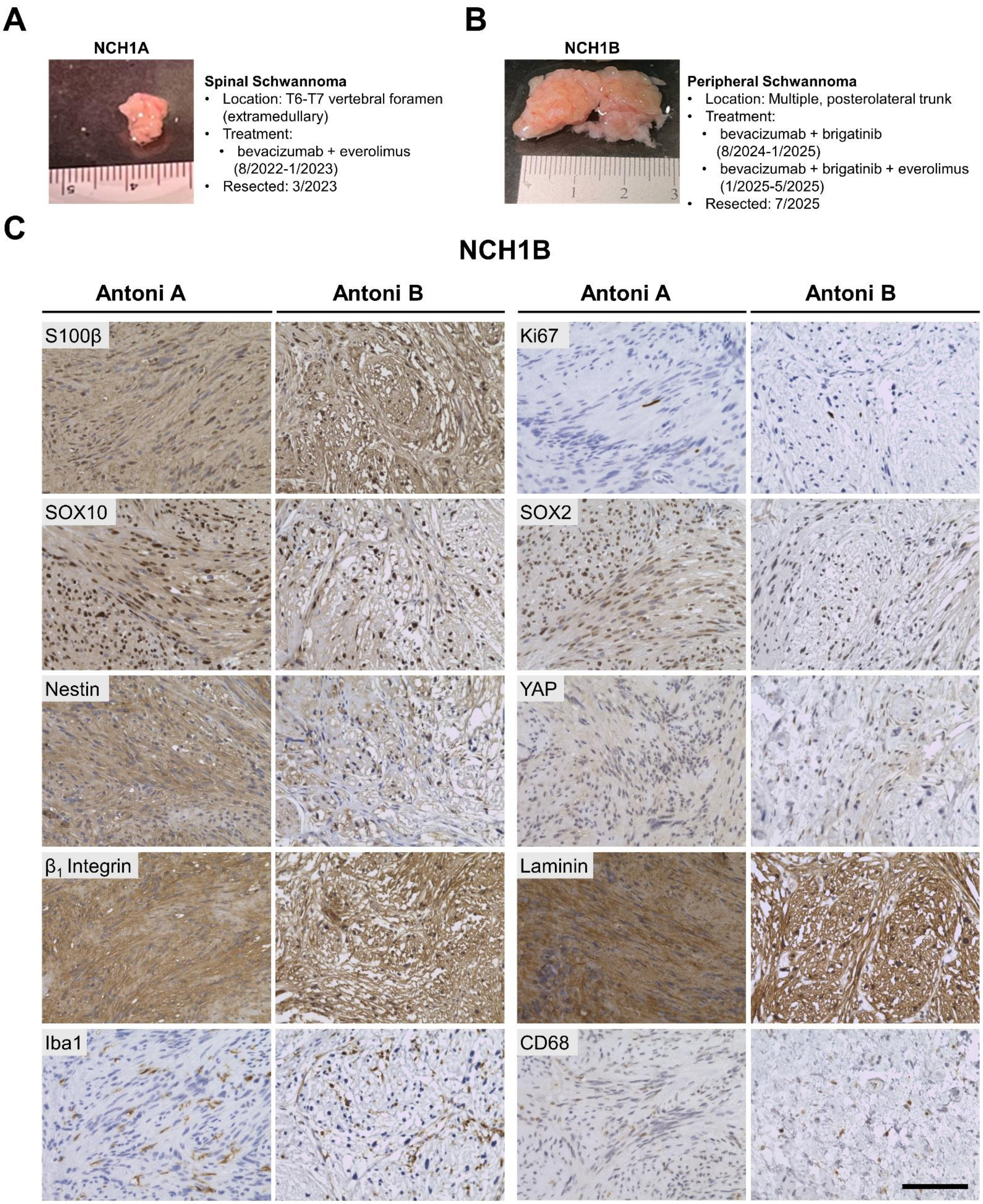

Figure S1. NCH1A and NCH1B Gross Histology and NCH1B Immunohistochemistry. Gross appearances of NCH1A **(A)** and NCH1B **(B)** tumor fragments before preservation. Rulers indicate centimeters. **(C)** Representative IHC staining of indicated markers in NCH1B. Scale bar = 100μm.

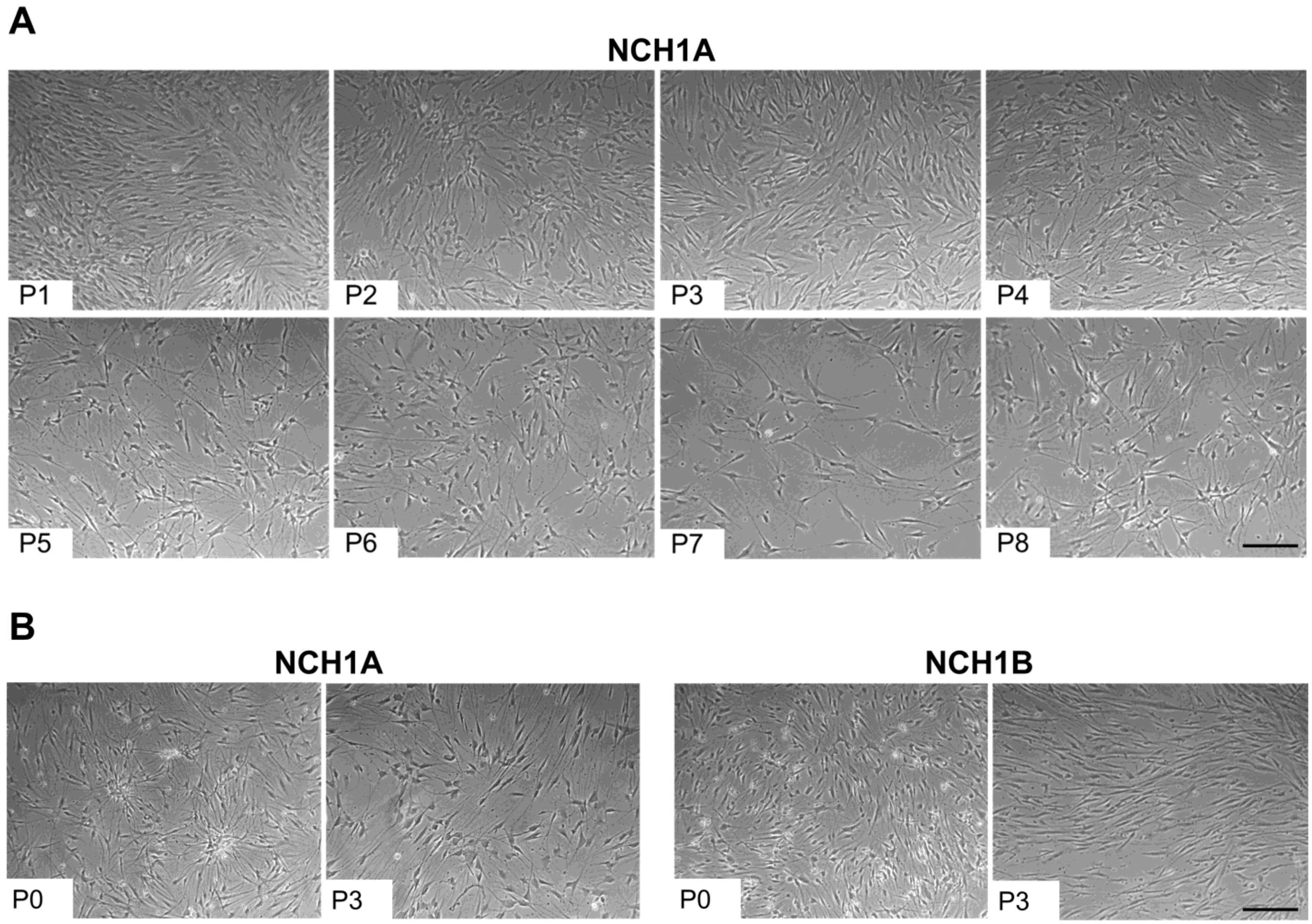

Figure S2. Morphological Appearance of Primary NCH1 Schwannoma Cells. (A) Phase contrast images of NCH1A cells over eight passages. **(B)** Phase contrast images of NCH1A and NCH1B cells immediately after isolation and at P3. Insets indicate passage number. Scale bars = 200 µm.

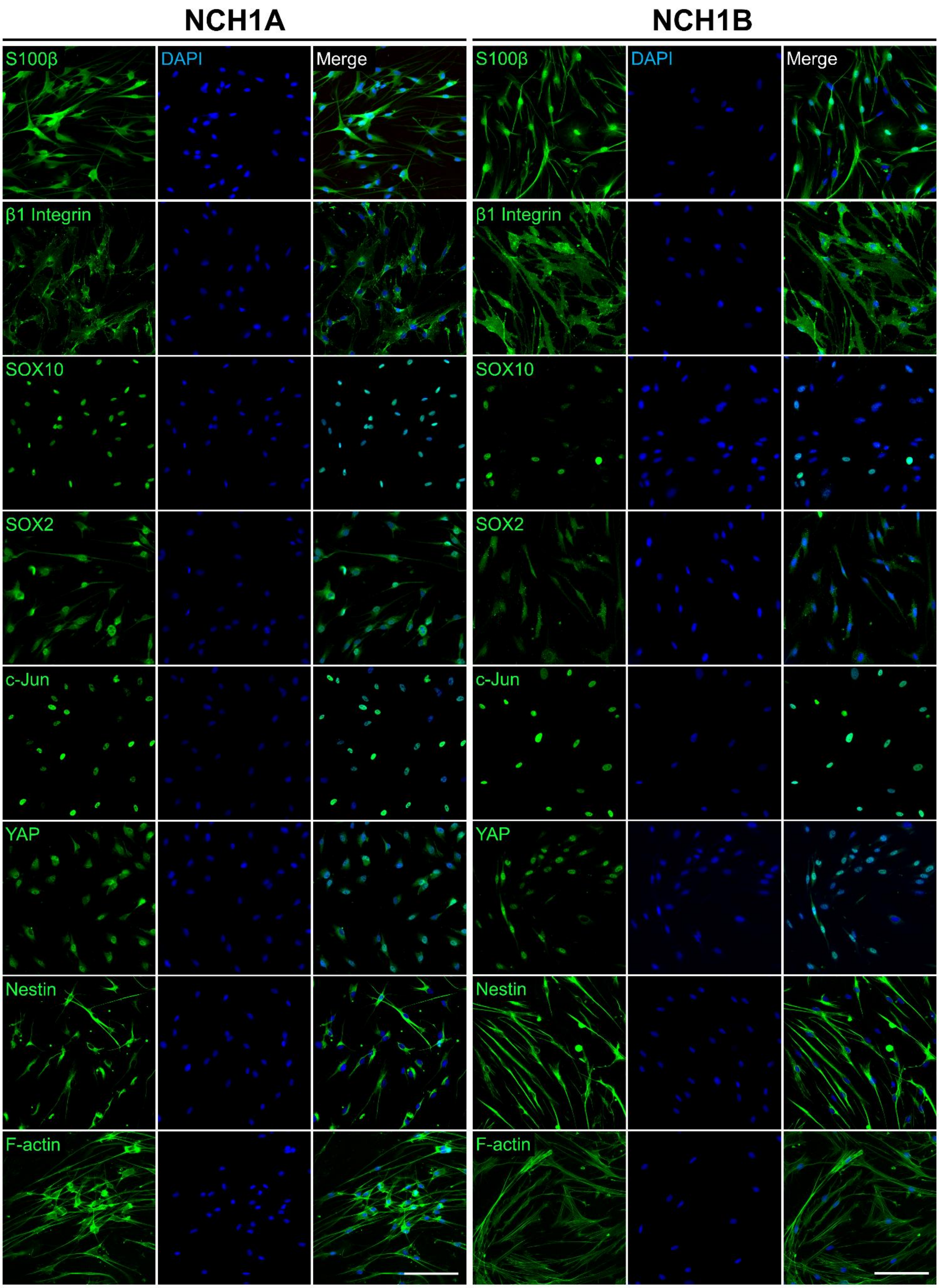

Figure S3. Individual Channels for NCH1 Schwannoma Cell Marker Expression. Scale bars = 100 μm.

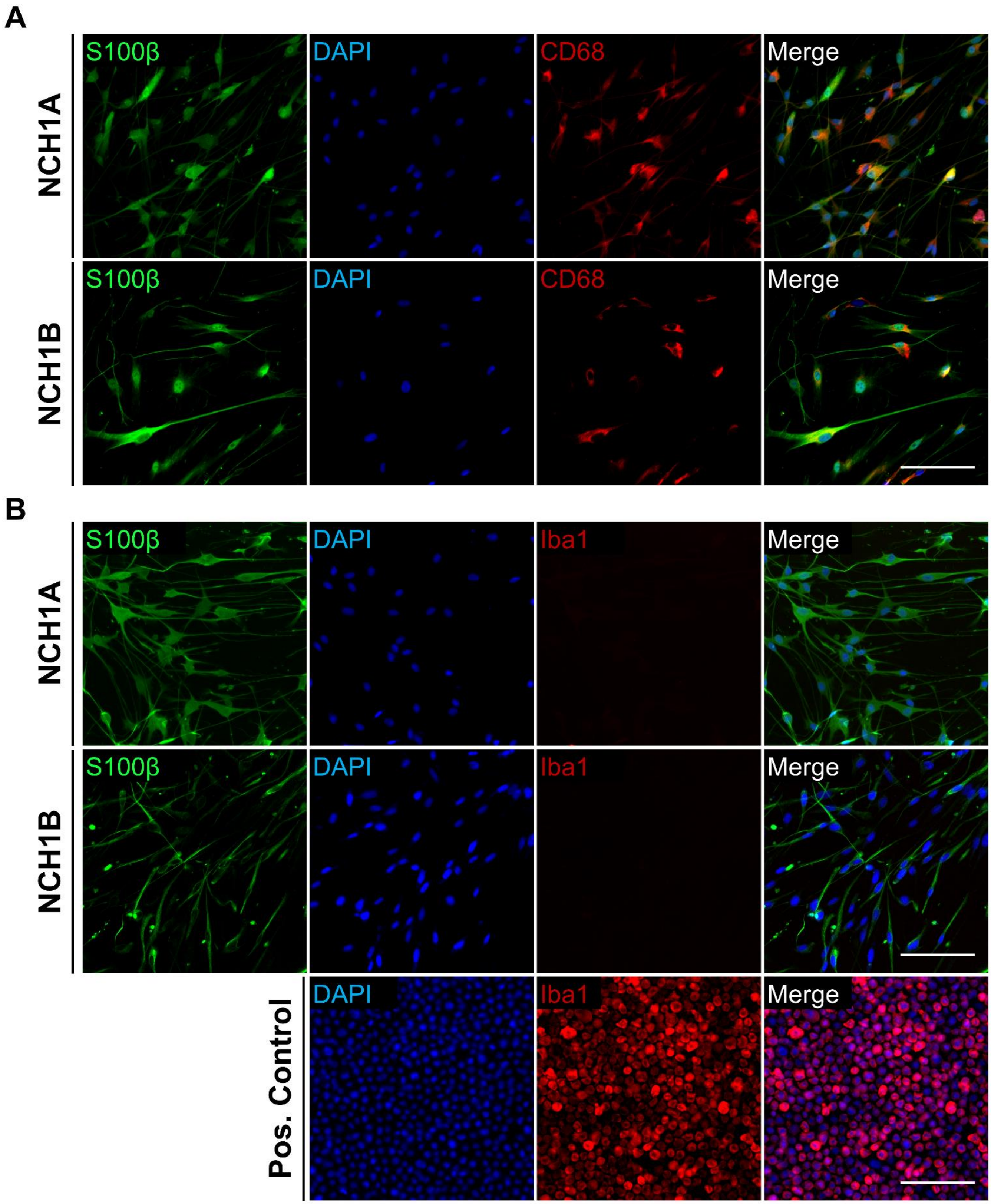

Figure S4. Expression of Immune Markers by NCH1 Schwannoma Cells. Co-staining for Schwann cell marker S100β (green) with macrophage markers **(A)** CD68 (red) and **(B)** Iba1 (red) in NCH1A and NCH1B. The acute monocytic leukemia cell line THP-1 was stained as positive control for Iba1. Suspended THP-1 cells were fixed using Cytoperm/Cytofix solutions (BD Biosciences), blocked with 10% normal goat serum, and incubated with anti-Iba1 antibody diluted in Cytoperm/Cytofix solution. Scale bars = 100 µm.

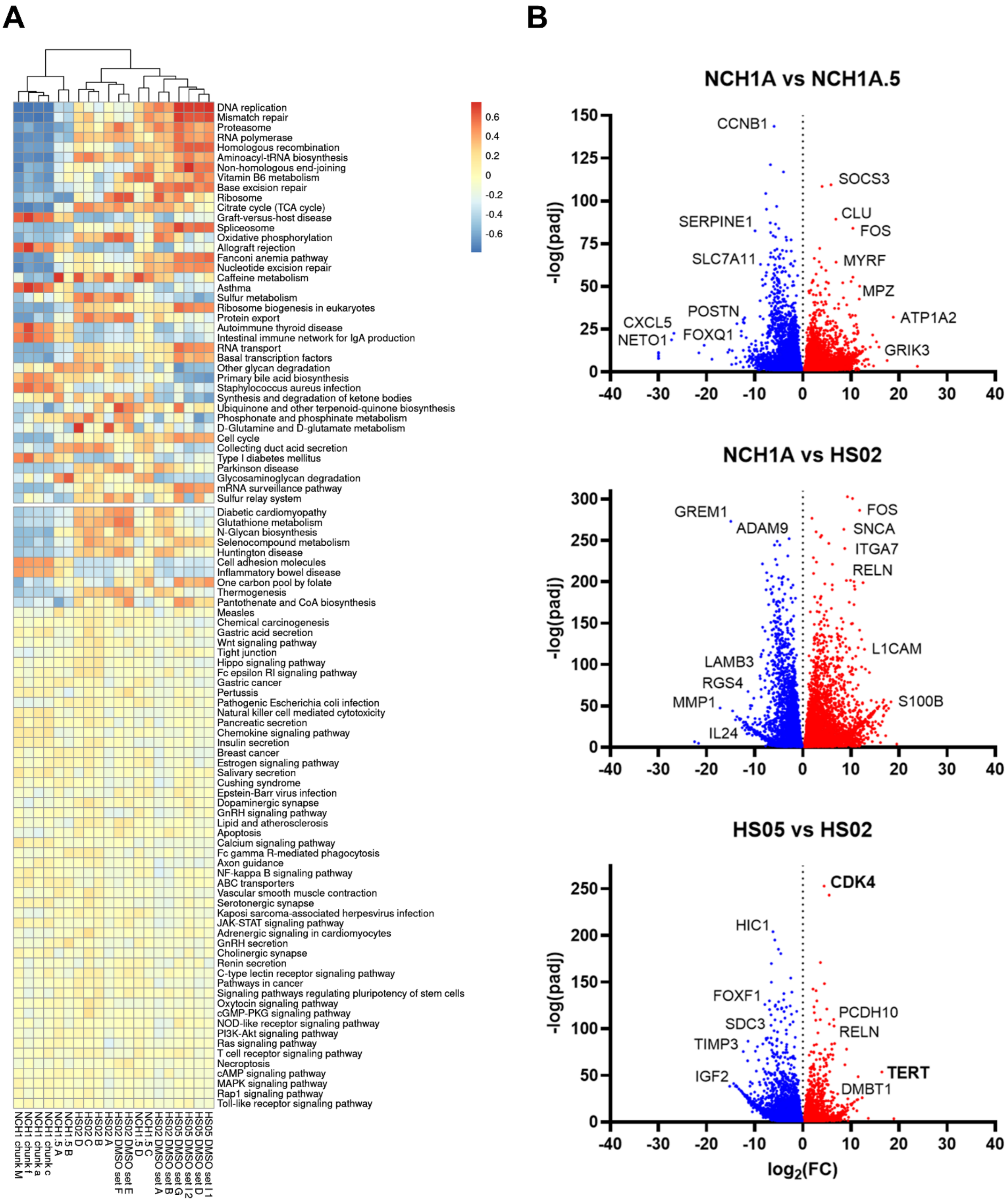

Figure S5. Expanded Analysis of NCH1A Tissues and Cultured Schwannoma Cells. (A) GSVA heatmap of the 50 most and least variable pathways across all samples from the KEGG 2021 Human gene set. Color scale indicates GSVA enrichment score. **(B)** Volcano plots depicting differential gene expression between NCH1A schwannoma tissues, primary cells (NCH1A.5), non-immortalized HS02 model cells, and immortalized HS05 model cells. *TERT* and *CDK4* genes bolded to indicate stable expression of immortalization factors in HS05 cells.

Table S2. Drugs Screened and Target Pathways

Drug (or Combination)	Target(s)
Fimepinostat	pan-HDAC, PI3K
Neratinib	pan-HER
Lapatinib	EGFR, HER2
Dasatinib	ABL, SFKs, FAK[34]
Neratinib + Dasatinib	Combination
Brigatinib	FAK[32], Fer[32], ALK
Crizotinib	FAK[27], ALK, c-MET
Cabozantinib	VEGFR2, c-MET, KIT, AXL
Trametinib	MEK1/2
Cobimetinib	MEK1/2
Selumetinib	MEK1/2
Everolimus	mTOR
Everolimus + Lapatinib	Combination
WP1066	JAK2, STAT3
BMS-986158	BET proteins (BRD4)
Lapatinib + BMS	Combination
Trametinib + BMS	Combination
Brigatinib + BMS	Combination

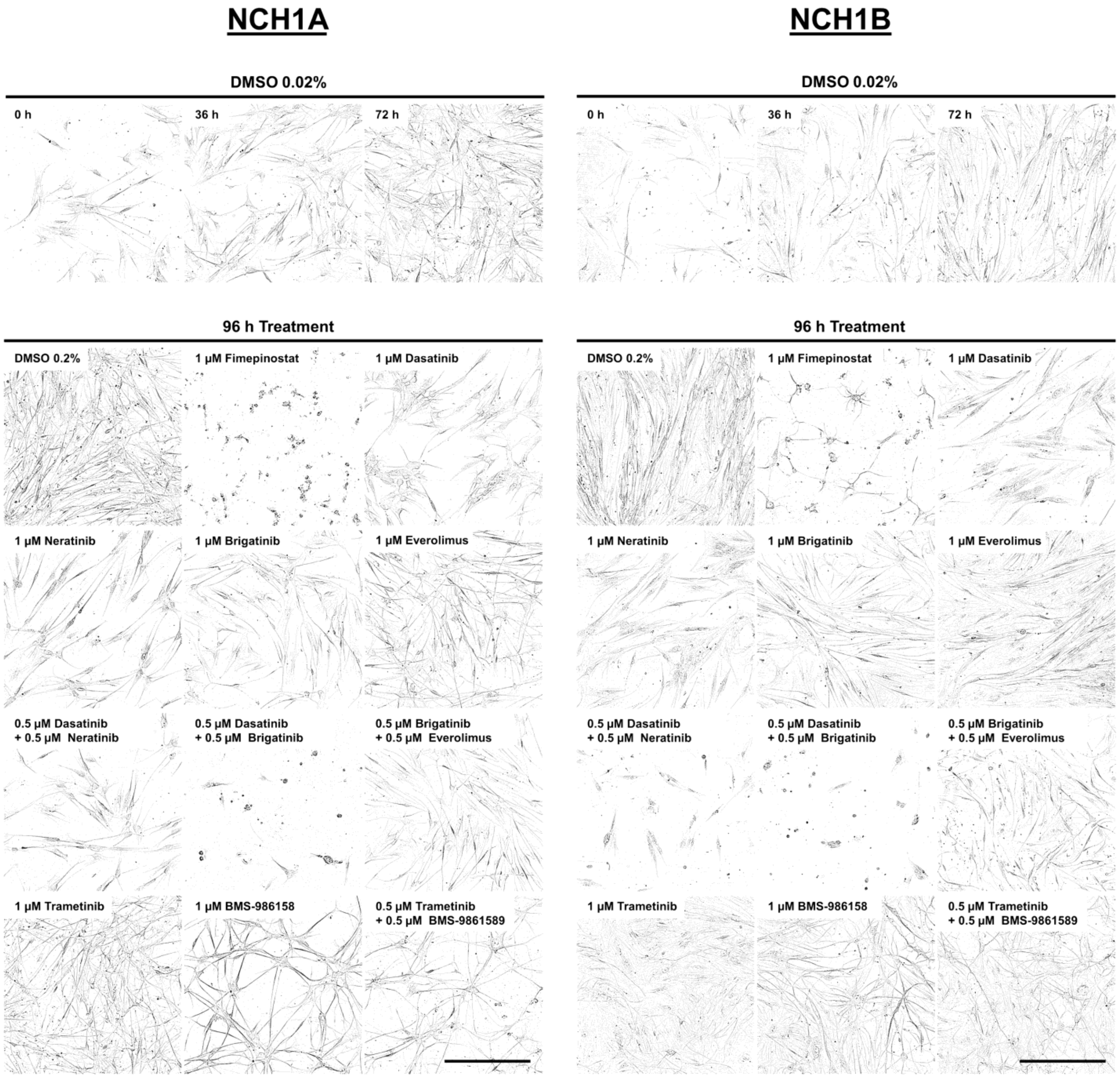

Figure S6. Morphological changes in NCH1 Schwannoma Cells with Indicated Drug Treatments. Representative images captured during single and combination drug screens by the Incucyte system at indicated times. Scale bars = 300µm.

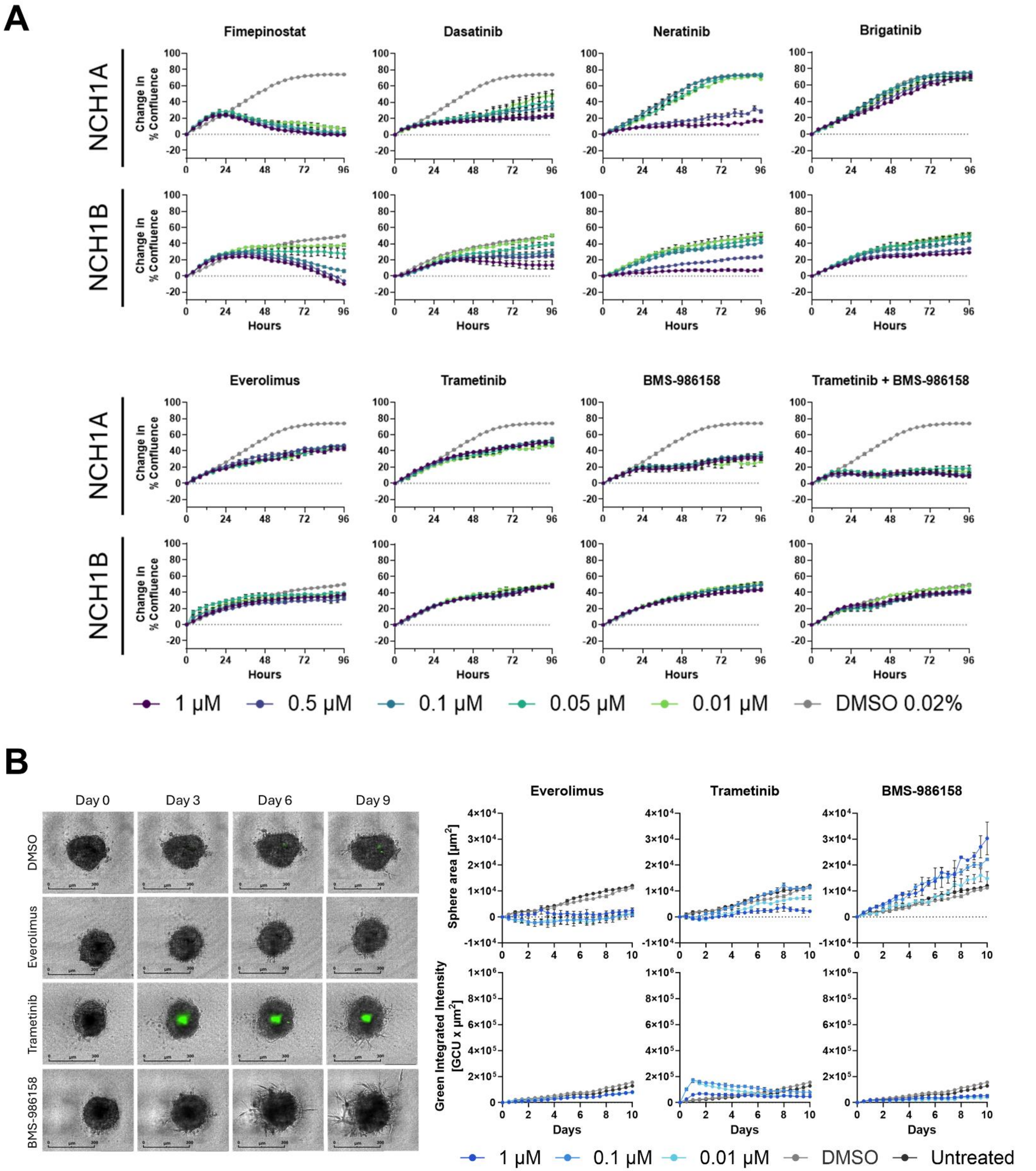

Figure S7. Confluence Changes and Spheroid Growth in NCH1 Schwannoma Cells with Indicated Drug Treatments. (A) Change in percent confluence (normalized to time of initial treatment) for adherent NCH1A and NCH1B cells treated with indicated drugs from primary screens. **(B)** Spheroid area and cell death (cleaved caspase 3/7; green integrated intensity normalized to time of initial treatment) measured during monotherapy screens with indicated drugs.

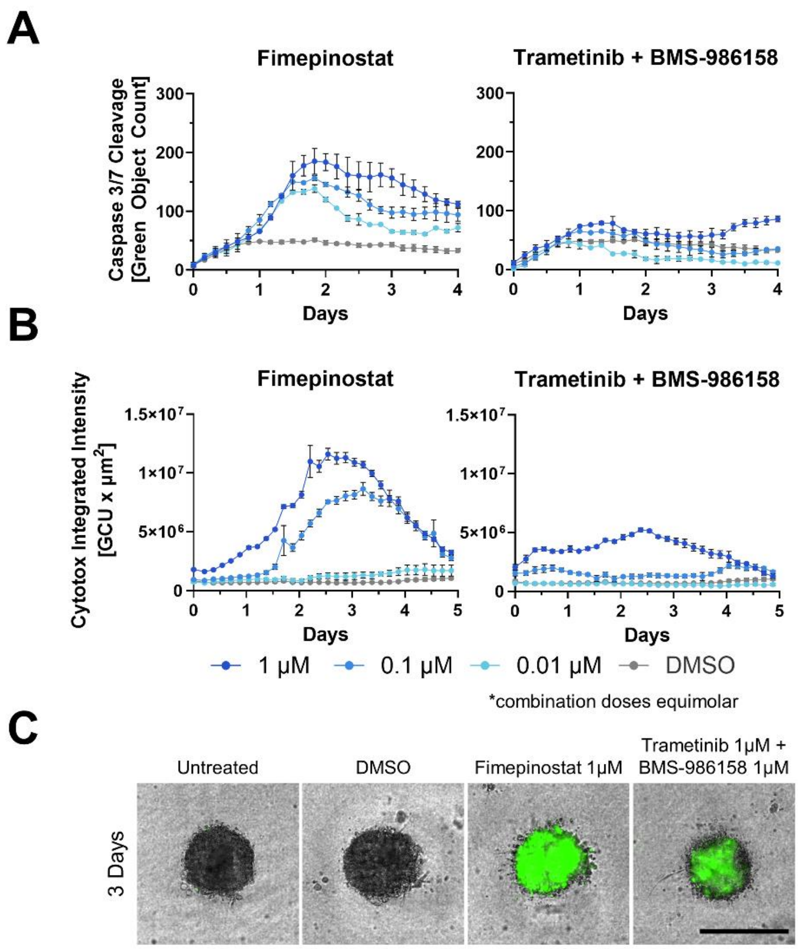

Figure S8. Cell Death Measured by Incucyte in NCH1A Adherent Cells and Spheroids with Fimepinostat and Trametinib Combined with BMS-986158. (A) Cleaved caspase 3/7 signal (green object count) detected in adherent NCH1A cells in one replicate of primary drug screens. **(B)** Cell death (green integrated intensity) in NCH1A spheroids detected using Cytotox reagent (Sartorius) over five days of treatment with indicated drugs. GCU = green calibrated unit. **(C)** Representative images of cell death (Cytotox signal) in NCH1A spheroids at three days untreated and treated with DMSO, fimepinostat, and the combination of trametinib with BMS-986158. Scale bar = 300 μm.

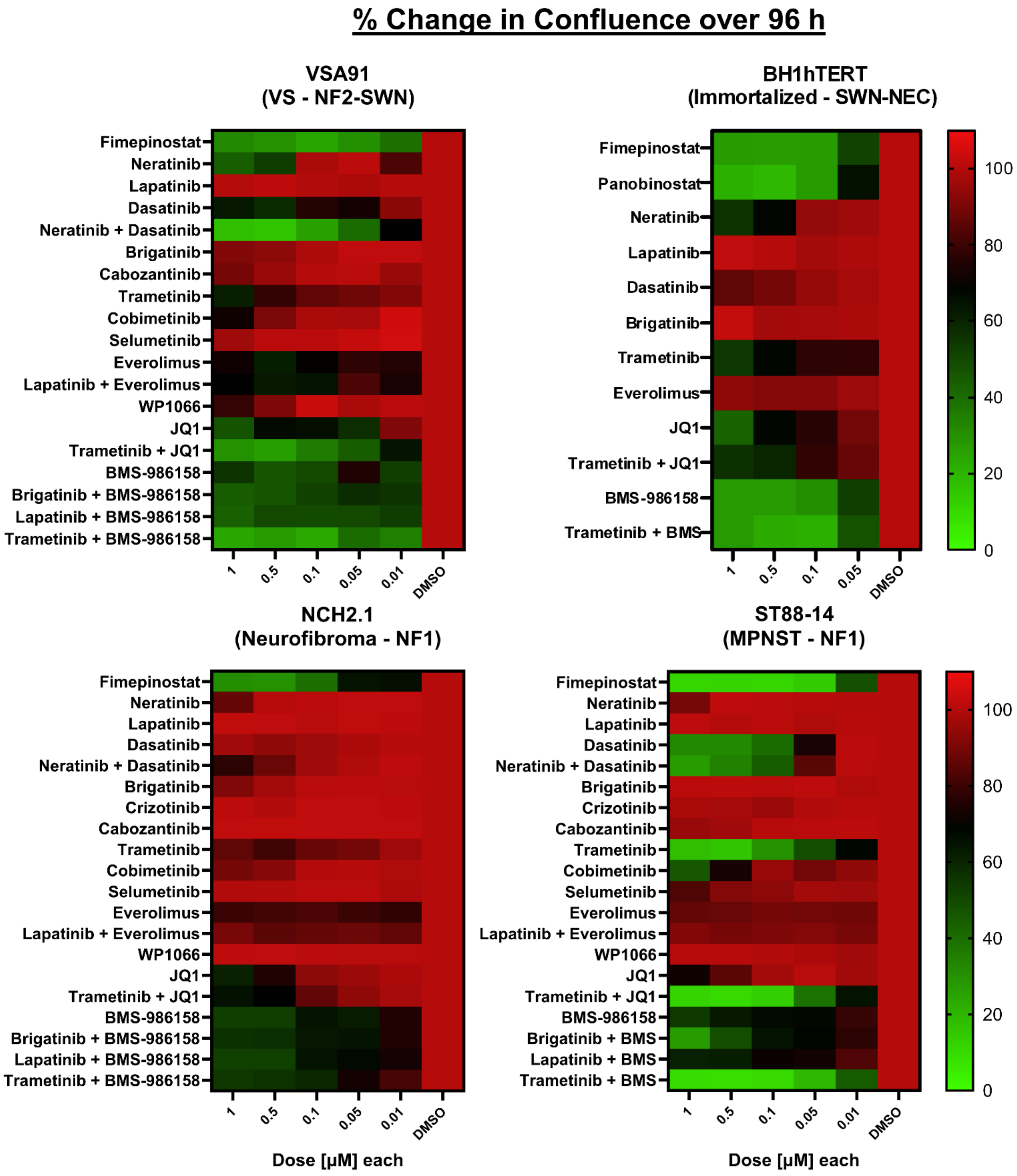

Figure S9. High-content Drug Screens Results in Other Nerve Tumor Cells. Heatmaps illustrating drug sensitivities for primary VS cells (VSA91), an *hTERT*-immortalized peripheral schwannoma cell line (BH1hTERT), primary cells from an NF1-related neurofibroma (NCH2.1), and a malignant peripheral nerve sheath tumor (MPNST) cell line (ST88). SWN-NEC = schwannomatosis not elsewhere classified. NF1 = neurofibromatosis type I.

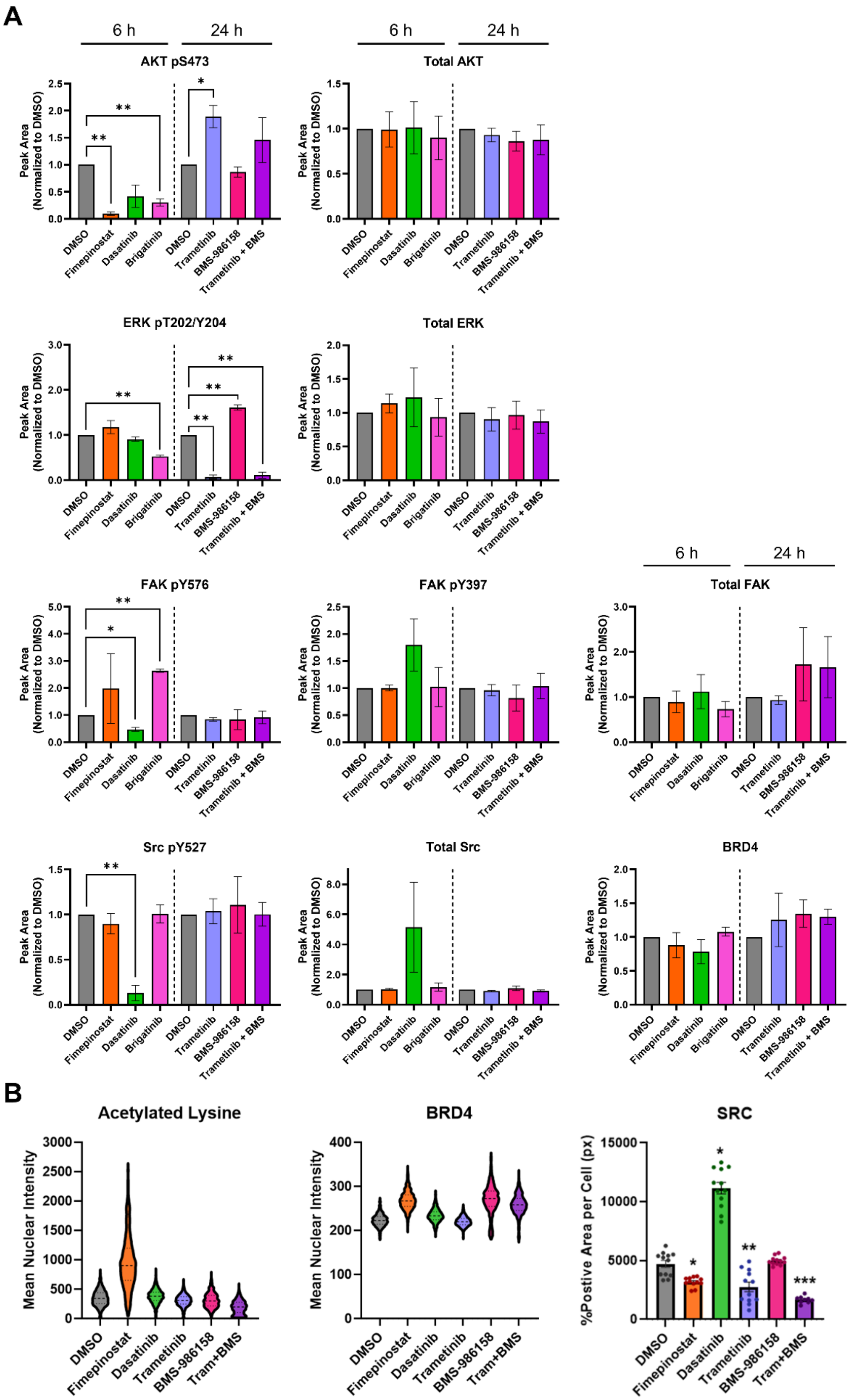

Figure S10. Jess Immunoblot and IF Quantification in NCH1A Schwannoma Cells. (A) NCH1A protein expression normalized to loading controls (β-actin or vinculin), then to respective DMSO-treated controls (6 h or 24 h treatment, n = 3 biological replicates). **(B)** Mean IF nuclear intensity and percent positive area (normalized to nuclear count) for indicated markers in NCH1A cells treated for 24 h (n = 12 regions of interest per condition). *p<0.05, **p<0.01 ***p<0.001.
